# Hypertension and cerebral blood flow in the development of Alzheimer's disease

**DOI:** 10.1002/alz.14233

**Published:** 2024-09-10

**Authors:** Dario Bachmann, Antje Saake, Sandro Studer, Andreas Buchmann, Katrin Rauen, Esmeralda Gruber, Lars Michels, Roger M. Nitsch, Christoph Hock, Anton Gietl, Valerie Treyer, for the Alzheimer's Disease Neuroimaging Initiative, Michael W. Weiner, John Q. Trojanowski, Leslie Shaw, Laurel Beckett, Paul Aisen, Ronald Petersen, John C. Morris, Richard J. Perrin, Arthur W. Toga, Clifford Jack, Robert C. Green, William Jagust, Andrew J. Saykin

**Affiliations:** ^1^ Institute for Regenerative Medicine University of Zurich Zurich Switzerland; ^2^ Department of Health Sciences and Technology ETH Zürich Zurich Switzerland; ^3^ Department of Geriatric Psychiatry Psychiatric Hospital Zurich Zurich Switzerland; ^4^ Neuroscience Center Zurich University of Zurich Zurich Switzerland; ^5^ Department of Neuroradiology Clinical Neuroscience Center, University Hospital Zurich Zurich Switzerland; ^6^ Neurimmune Zurich Switzerland; ^7^ Department of Nuclear Medicine University Hospital of Zurich, University of Zurich Zurich Switzerland; ^8^ University of California San Francisco USA; ^9^ University of Pennsylvania Pennsylvania USA; ^10^ University of California Davis USA; ^11^ University of Southern California California USA; ^12^ Mayo Clinic Rochester Minnesota USA; ^13^ Washington University St. Louis USA; ^14^ Brigham and Women's Hospital/Harvard Medical School Massachusetts USA; ^15^ University of California Berkeley USA; ^16^ Indiana University Indiana USA

**Keywords:** Alzheimer's disease, amyloid pathology, arterial spin labeling, cerebral perfusion, regional white matter hyperintensities, small vessel disease, tau pathology

## Abstract

**INTRODUCTION:**

We investigated the interactive associations between amyloid and hypertension on the entorhinal cortex (EC) tau and atrophy and the role of cerebral blood flow (CBF) as a shared mechanism by which amyloid and hypertension contribute to EC tau and regional white matter hyperintensities (WMHs).

**METHODS:**

We analyzed data from older adults without dementia participating in the Add‐Tau study (NCT02958670, *n* = 138) or Alzheimer's Disease Neuroimaging Initiative (ADNI) (*n* = 523) who had available amyloid‐positron emission tomography (PET), tau‐PET, fluid‐attenuated inversion recovery (FLAIR), and T1‐weighted magnetic resonance imaging (MRI). A subsample in both cohorts had available arterial spin labeling (ASL) MRI (Add‐Tau: *n* = 78; ADNI: *n* = 89).

**RESULTS:**

The detrimental effects of hypertension on AD pathology and EC thickness were more pronounced in the Add‐Tau cohort. Increased amyloid burden was associated with decreased occipital gray matter CBF in the ADNI cohort. In both cohorts, lower regional gray matter CBF was associated with higher EC tau and posterior WMH burden.

**DISCUSSION:**

Reduced cerebral perfusion may be one common mechanism through which hypertension and amyloid are related to increased EC tau and WMH volume.

**Highlights:**

- Hypertension is associated with increased entorhinal cortex (EC) tau, particularly in the presence of amyloid.
- Decreased cortical cerebral blood flow (CBF) is associated with higher regional white matter hyperintensity volume.
- Increasing amyloid burden is associated with decreasing CBF in the occipital lobe.
- MTL CBF and amyloid are synergistically associated with EC tau.

## 1 BACKGROUND

Hypertension is a major contributor to subclinical brain damage that leads to cognitive dysfunction in older individuals through multifaceted mechanisms.^1^ One pathway by which hypertension increases the risk of cognitive decline is mediated by vascular brain damage such as white matter lesions. A second, less comprehended, pathway may be related to Alzheimer's disease (AD) pathology, either by directly increasing amyloid beta (Aβ) pathology or interactively with Aβ increasing tau pathology.^2^ Both pathways may converge in the medial temporal lobe (MTL) and cause atrophy of these critical structures that are essential for episodic memory. However, these pathways are not completely independent of each other, and a better understanding of the interplay between hypertension and AD pathology is essential for effective treatment and risk stratification.

A notable commonality between hypertension and AD pathology emerges when one considers the entorhinal cortex (EC) within the MTL. The EC is a consistent site of neuronal pathology and volume loss in aging and the earliest stages of AD.^3^ Previous studies revealed Aβ‐independent associations between vascular diseases and EC tau^4^, ^5^ as well as synergistic effects of vascular risk factors and Aβ pathology on MTL lobe tau^2^ and atrophy.^6^ Hypertension is also a major risk factor for white matter hyperintensities (WMHs),^7^ which themselves have been linked to tau pathology,^8^, ^9^ reduced MTL volume,^10^ and atrophy of the EC.^11^ Hence, it is reasonable to speculate that the correlation between WMHs and EC tau or atrophy may be attributed to shared risk factors, such as hypertension, influencing both processes. However, while the strongest associations between hypertension and WMH burden have been observed within deep frontal regions,^12^, ^13^ an association between WMHs and EC atrophy is most notable when WMHs are examined in the occipital and parietal lobes.^10^, ^11^, ^14^

A common mechanism underlying the aforementioned processes may be altering blood supply to these regions. Both Aβ pathology and chronic hypertension may lead to decreased cerebral blood flow (CBF), and chronic ischemia is one major contributor to the development of WMHs.^15^, ^16^, ^17^, ^18^ Furthermore, studies in rodent models of chronic cerebral hypoperfusion reported increased levels of soluble tau,^19^, ^20^ suggesting that chronic ischemia can induce tau pathology. Thus, a decline in CBF may not only act as an additional cause of brain damage in AD but actively contribute to the exacerbation of the supposed core processes driving the disease.

The aim of this study was to investigate the potentially interacting pathways through which hypertension and AD pathology may contribute to EC atrophy and memory decline. We hypothesized that some associations among hypertension, WMHs, and EC tau vary depending on the presence of Aβ pathology and the lobar region in which WMH burden is assessed. Furthermore, we hypothesized that altered CBF is a common mechanism by which Aβ and hypertension contribute to EC tau and WMH burden.

## 2 METHODS

### 2.1 Participants

We analyzed data from 141 participants without dementia from the baseline observation of a longitudinal clinical trial conducted at the University of Zurich, Switzerland (Add‐Tau study: NCT02958670). All participants were volunteers who had previously participated in studies involving Aβ imaging and were initially recruited through newspaper advertisements. All participants had Aβ‐positron emission tomography (PET) and tau‐PET scans available. Three participants were excluded because of errors during the tau‐PET acquisition (*n* = 2) or insufficient quality of the T1‐weighted image (*n* = 1), leading to a final sample of 138 individuals. After excluding participants with insufficient quality of the arterial spin labeling (ASL) scan (*n* = 3), CBF estimates were available for a subsample of 78 individuals. Participants were diagnosed as cognitively unimpaired or having mild cognitive impairment (MCI) according to consensus criteria.^21^

As the Aβ burden of the Add‐Tau cohort, particularly in the subsample with available ASL magnetic resonance imaging (MRI), was relatively low, we extended our results to a population in more advanced Aβ stages using data from the Alzheimer's Disease Neuroimaging Initiative^22^ (ADNI). We included 523 participants without a diagnosis of dementia with available Aβ‐PET and tau‐PET as well as memory assessment, three‐dimensional (3D) fluid attenuated inversion recovery (FLAIR), and T1‐weighted structural MRI within 6 months of the tau‐PET scan. After the exclusion of participants whose ASL scan did not pass quality control, CBF estimates were available for a subsample of 89 individuals. Participants were diagnosed by the ADNI investigators as cognitively unimpaired (Mini‐Mental State Examination [MMSE] score ≥24, Clinical Dementia Rating [CDR] of 0, and normal memory function on education‐adjusted Wechsler Memory Scale II) or MCI (MMSE score ≥24, CDR of 0.5, and objective memory impairment on education‐adjusted Wechsler Memory Scale II). Demographic information, apolipoprotein E (*APOE*) ε4 carrier status, cognitive data, clinical information, and imaging data were downloaded from the ADNI data repository (http://adni.loni.usc.edu). Figure S1 summarizes the sample sizes for the analyses per cohort. The Add‐Tau study was approved by the ethics committee of the canton of Zurich and the competent authority (Swissmedic). The ADNI study was approved by all sites’ respective review boards. Written informed consent was obtained from all participants.

### 2.2 MRI and PET acquisition

#### 2.2.1 Add‐Tau study

We used the following MRI scans acquired on a 3T General Electric (GE) scanner: T1‐weighted fast spoiled gradient recalled acquisition (0.5 × 0.5 × 0.5 mm voxel size), 3D Cube FLAIR (0.48 × 0.48 × 0.6 mm voxel size), and 3D fast‐spin‐echo stack‐of‐spirals pseudo‐continuous ASL (PCASL; 1.9 × 1.9 × 3 mm reconstructed voxel size). PCASLs were acquired with post‐labeling delay = 2.025 s, labeling duration = 1.450 s, repetition time (TR) = 5.07 s, echo time (TE) = 11.5 ms, and number of excitations (NEX) = 3. FLAIR and ASL images were acquired at the tau‐PET scan acquisition visit. T1‐weighted images were acquired at a maximum of 6 months before the tau‐PET.

Aβ plaque deposition was assessed using a radiotracer, [18F]‐flutemetamol (*n* = 99) or [11C]‐Pittsburgh Compound‐B (*n* = 39), and deposition of tau was assessed using [18F]‐flortaucipir. Detailed acquisition and processing procedures of Aβ‐PET and tau‐PET scans were described previously.^23^ The median (interquartile range [IQR]) duration from Aβ‐PET to the tau‐PET/ASL acquisition was 1.3 (0.9 to 2) years.

**RESEARCH IN CONTEXT**

1. **Systematic review**: Relevant articles were identified using Google Scholar and by reviewing cited works. White matter disease is commonly seen in Alzheimer's disease (AD) and has been associated with amyloid pathology, tau pathology, entorhinal cortex atrophy, and memory decline. However, the role of hypertension and cerebral blood flow in these associations has not been thoroughly explored.
2. **Interpretation**: Hypertension has a multifaceted influence on the development of AD dementia. Reduced cerebral perfusion may be one common mechanism through which hypertension and amyloid relate to tau pathology and white matter damage. Preventing hypertension may reduce the risk of Alzheimer's dementia through both AD pathology‐dependent and independent mechanisms.
3. **Future directions**: The relationship between hypertension and AD pathology should be investigated in longitudinal studies. Future studies should investigate whether controlling blood pressure also reduces AD pathology.

#### 2.2.2 ADNI

MRI and PET acquisition and processing details can be found online (http://adni.loni.usc.edu). We used 3D Cube FLAIR (1 × 1 × 1.2 mm voxel size) acquired on SIEMENS (*n* = 327), GE (*n* = 126), or Philips (*n* = 70) scanners. 3D PCASL (1.9 × 1.9 × 4 mm reconstructed voxel size) images were acquired on 3T GE scanners with post‐labeling delay = 2.025 s, labeling duration = 1.450 s, TR = 4.89 s, TE = 11.5 ms, and NEX = 3. Aβ plaque deposition was assessed using [18F]‐florbetapir (*n* = 293) or [18F]‐florbetaben (*n* = 230), and tau deposition was assessed using [18F]‐flortaucipir. We selected the Aβ‐PET scan closest in time to either the tau‐PET scan or the ASL MRI scan for those with ASL MRI data, but no restrictions were imposed on the time between the two acquisitions to avoid additional reductions in sample size. The median (IQR) time difference between Aβ‐PET and ASL was 19 (0 to 35) days and 2 days (−5 to 15) between Aβ‐PET and tau‐PET.

### 2.3 MRI and PET preprocessing

#### 2.3.1 Add‐Tau study

T1‐weighted images were segmented and parcellated using FreeSurfer version 7.1.1 to define regions of interest (ROIs) and extract EC thickness. FreeSurfer parcellation of each participant was visually inspected for accuracy, and segmentation errors were manually corrected. WMHs were segmented on FLAIR images using the lesion prediction algorithm as implemented in the lesion segmentation toolbox (LST) toolbox (www.statistical‐modelling.de/lst.html) for the statistical parametric mapping (SPM) software as previously described.^24^ Lesion masks were created by binarizing lesion probability maps at a threshold of 0.65. This threshold was selected after applying different thresholds to 20 randomly selected subjects and visually expecting the generated binarized lesion masks for accuracy. Finally, lesion masks were visually inspected and manually corrected if necessary. For seven participants, WMH volume estimation was not possible due to insufficient quality of the FLAIR image (treated as “missing value”; see Section 2.7). WMH volume was extracted in each individual's native FLAIR space for occipital and parietal lobe and the deep frontal white matter. The deep frontal ROI included all voxels in the frontal lobe that are closer to the neocortex than to the wall of the lateral ventricles.^25^ Occipital and parietal ROIs were selected as previous studies linked higher WMH volume within these regions to Aβ pathology,^26^ EC atrophy,^11^ and progression to AD.^27^ To confirm that the observed associations with WMHs are region‐dependent, we included the deep frontal ROI for which we anticipated a robust association with hypertension but no correlation with AD pathology.^12^, ^13^, ^25^ We calculated relative WMH (rWMH) volume by normalizing each participant's WMH volume in each ROI with total intracranial volume. rWMHs were log‐transformed to reduce skewness. Total intracranial volume showed strong correlations with regional white matter volumes (Figure S2). Therefore, using total intracranial volume as opposed to regional white matter volume to normalize WMH burden is unlikely to significantly affect the reported results. CBF maps were quantified following a single‐compartment model approach and the recommendations outlined in the ASL consensus paper.^28^ A detailed description of the CBF quantification is provided in the Supplementary Methods. In brief, perfusion‐weighted images were directly provided by the scanner and were co‐registered to each participant's FreeSurfer‐processed T1‐weighted image using a boundary‐based cost function.^29^ Using the inverse transformation, gray and white matter probability maps were transformed to the ASL space to correct perfusion‐weighted images for partial volume effects using a voxel‐wise local linear regression algorithm.^30^ After M0 division, CBF maps were transformed to the FreeSurfer space to obtain mean gray matter regional CBF from the occipital, parietal, frontal, and medial temporal lobe regions. These regions were created by combining the corresponding FreeSurfer‐segmented ROIs. For the MTL region, we combined EC, parahippocampal cortex, hippocampus, and amygdala ROIs. To adjust for variations in ASL signal associated with scanner and physiological variance, we computed relative CBF (rCBF) by normalizing each participant's CBF values with the mean CBF value in the brainstem. The brainstem was selected as the reference region as it was available in both the Add‐Tau and ADNI cohorts and is typically not substantially affected in early disease stages.^31^ We obtained similar results when the precentral gyrus^32^, ^33^ was used as an alternative reference region (Figures S3 and S4). Absolute CBF values in milliliters per 100 g per minute (mL/100 g/min) are reported in Table S1. Due to erroneous registration of the CBF map to the structural scan, we excluded one participant's CBF measurement for the frontal lobe (treated as “missing value”; see Section 2.7).

For Aβ‐PET, we calculated Centiloids using PMOD NeuroTool (version 3.9, PMOD Technologies LLC) to increase comparability across Aβ‐PET methods and tracers.^34^ Partial volume effect corrected (PVC) EC tau‐PET was normalized by mean uptake in the inferior cerebellar cortex to calculate the standardized uptake value ratio (SUVR).^23^

#### 2.3.2 ADNI

The PVC CBF values for FreeSurfer ROIs, PVC EC tau uptake, EC thickness, and Centiloid values were obtained from relevant spreadsheets downloaded from the ADNI Image and Data Archive website. Comprehensive image processing details can be accessed online. The tau‐PET data were normalized by the inferior cerebellar cortex. The segmentation of WMHs followed the methodology outlined for the Add‐Tau cohort. Details can be found in the Supplementary Methods. The thresholds applied to the lesion probability maps generated by the LST varied depending on the scanner manufacturer. For FLAIR scans acquired on SIEMENS or Philips scanners, we used a threshold of 0.35 on the lesion probability maps. If the FLAIR scan was acquired on a GE scanner, a threshold of 0.50 was applied. Lesion masks were visually assessed for accuracy and corrected as necessary. As further described in the Supplementary Methods, individuals scanned on a Philips scanner had considerably higher WMH lesion volume compared to individuals scanned on a GE or SIEMENS scanner. To ensure that our results are not influenced by systemic WMH volume differences among manufacturers due to the LST, we excluded individuals scanned on a Philips scanner in a sensitivity analysis. Note that for participants with ASL data, FLAIR images were exclusively acquired on a GE scanner. The rWMH for the occipital, parietal, and deep frontal ROIs was calculated by normalizing each with total intracranial volume and then applying a log transformation, similar to the Add‐Tau cohort. To determine rCBF for the occipital, parietal, and frontal lobes, we averaged the CBF values from the corresponding FreeSurfer ROIs and normalized them by the mean CBF in the brainstem.^31^ See Figures S3 and S4 for results using the precentral gyrus as the reference region.

### 2.4 Amyloid‐PET status

To maintain consistency in the analyses across cohorts, we used a shared threshold for Aβ+ status, defined as Centiloid > 12, which facilitates direct comparisons between the ADNI and Add‐Tau cohorts. We adhered to this relatively low threshold for Aβ positivity because many participants in the Add‐Tau cohort are in the early stages of Aβ accumulation, and we wanted to avoid further reducing the sample size in the Aβ+ group. A Centiloid value of 12 has previously been identified as an indicator of the transition from the absence of Aβ pathology to the presence of subtle pathology.^35^

### 2.5 Hypertension and blood pressure measurement

For the Add‐Tau cohort, hypertension status was self‐reported during the assessment of the participant's medical history. Furthermore, a single blood pressure measurement was collected while in a supine position after resting for a duration of 5 min. In the ADNI cohort, participants were classified as hypertensive if they reported using antihypertensive medication at the time of the tau‐PET/ASL scan. ADNI participants reporting no antihypertensive medication use but who had blood pressure measures above stage 2 hypertension (>160/100 mmHg) at the time of the tau‐PET/ASL scan were also classified as hypertensive (*n* = 20 in the total cohort; *n* = 8 in ASL subsample). Blood pressure measurements were conducted with participants in a seated position.

### 2.6 Cognitive assessment

We used an episodic memory composite score as a decline in episodic memory represents one of the initial cognitive indicators of AD dementia and shows associations with tau‐PET burden early in the disease process.^24^, ^36^ For the Add‐Tau cohort, the episodic memory composite score was based on seven cognitive tests: Consortium to Establish a Registry for Alzheimer's Disease (CERAD) words [learning, recall, and recognition], German version of the Rey Auditory Verbal Learning Test (RAVLT) [learning, late recall, and recognition], and CERAD figures [recall]. We converted each individual test score to *z*‐scores using the mean and standard deviation of the cohort, then averaged the *z*‐scores to create the episodic memory composite score.^37^ For ADNI, we used the memory cognitive composite score developed by the Alzheimer's Disease Sequencing Project Phenotype Harmonization Consortium (ADSP‐PHC).^38^ This score includes tasks assigned to the memory domain from the following cognitive tests: Wechsler Memory Scale‐Revised, RAVLT, Alzheimer's Disease Assessment Schedule‐Cognition, MMSE, and Montreal Cognitive Assessment.

### 2.7 Statistical analysis

Participant characteristics between Aβ− and Aβ+ participants within the Add‐Tau and ADNI cohorts were compared using Wilcoxon tests for continuous variables and *χ* ^2^ tests for categorical variables. Differences in hypertension prevalence were also examined using logistic regression models adjusted for age, sex, and *APOE* ε4. Regional rWMH burden differences were further assessed using linear regression models, with Aβ status as the predictor and age, sex, APOE ε4, and hypertension as covariates.

We used structural equation modeling to examine hypothesized models and explore group differences in multigroup analyses. Considering previous studies, outlined in the introduction, which suggest that hypertension and WMH burden are associated with EC tau and EC thickness, we specified paths from both hypertension and regional rWMH burden to EC tau, EC thickness, and episodic memory. Furthermore, we also considered that effects of hypertension might be mediated through rWMH burden. Aligning with the hypothesis that regional rWMH and EC tau pathology are linked through shared risk factors, we specified a covariance between rWMH burden and EC tau. We specifically focused on group differences between Aβ− and Aβ+ participants as paths may differ depending on the presence or absence of Aβ pathology.^2^, ^6^ Expanding on the findings of our initial model, we designed two subsequent models to investigate whether the association between hypertension and Aβ burden with regional rWMH volume and EC tau was mediated through altered regional rCBF. First, we examined the hypothesized paths to regional rWMH burden, specifying Aβ burden and rCBF as potential mediators between hypertension and rWMH. Additionally, rCBF served as a potential mediator in the association between Aβ and rWMH. Second, we used a moderated mediation model, with MTL rCBF as the mediator, EC tau as the outcome variable, and Aβ status as the moderator. Finally, to increase comparability with studies that used systolic blood pressure (SBP) measurements rather than relying on a hypertension diagnosis, we repeated the foregoing analyses using the earliest available SBP measurements, assessed during screening visits. All structural equation models were estimated separately for the Add‐Tau and ADNI cohorts.

We investigated differences between Aβ− and Aβ+ groups in the specified models by systematically constraining the path of interest to be equal, that is, the path coefficient was the same in both groups. Using a likelihood ratio test, the fit of the model that imposed an equality constraint was then compared to the fit of a baseline model where all parameters were allowed to be freely estimated in both groups. As recommended for multigroup structural equation modeling, these comparisons were adjusted for Type I error inflation.^39^, ^40^ A false discovery rate (FDR)‐corrected *p*‐value ≤ .05 indicated that the constrained model exhibited a significantly poorer fit than the unconstrained model, implying that the path should be estimated separately for each group. A final model was then estimated, allowing coefficients on paths to vary between groups if significant decreases in model fit were observed upon constraining the path. Paths with no observed group differences were assumed to be equal and estimated using the combined data.

Models were controlled for age at tau‐PET, sex, *APOE* ε4 carrier status, and the time difference between Aβ and tau‐PET acquisition for paths involving Aβ burden. Episodic memory performance was additionally adjusted for the number of previous neuropsychological assessments (only Add‐Tau cohort) and education. Model fit indices for all main models are presented in Table S2. Given the small degrees of freedom in certain models, we report the *χ* ^2^ test statistic, standardized root mean square residual (SRMR), and comparative fit index (CFI), as these metrics are less susceptible to the effects of degrees of freedom.^41^

We conducted the following sensitivity analyses. First, a significant Mahalanobis distance (*p *< .001) was used to define outliers in each model. Models were then refitted with outliers excluded. Second, we repeated the analysis after restricting the effects of covariates to be the same for the Aβ− and Aβ+ groups, as group differences may be influenced by covariates that also have group‐specific effects. The effects of the *APOE* ε4 variable were not constrained, as differences between Aβ− and Aβ+ groups on paths involving APOE ε4 may be expected. Third, models involving CBF were adjusted for education, body mass index (BMI), antihypertensive medication use (only Add‐Tau cohort), and MMSE score as an indicator of disease stage.

Analyses were performed in R version 4.2.2. Model assumptions were checked using the olsrr package (version 0.5.3). Models were estimated using robust standard errors and a scaled test statistic (*mlr* estimator) in the lavaan package (version 0.6.11.1676). All continuous variables were standardized prior to model entry. Full information maximum likelihood estimation was used to handle missing values.

## 3 RESULTS

### 3.1 Cohort characteristics

Cohort characteristics for the Add‐Tau and ADNI cohorts are shown in Table 1. In the Add‐Tau cohort, 36 (26%) out of 138 participants were Aβ+. In the ADNI cohort, 234 out of 523 participants (44.7%) were Aβ+. In the Add‐Tau cohort, Aβ+ participants had higher deep frontal WMH volume (*p* = .042) and were more likely to have hypertension compared to Aβ− participants (*p* = .002). Aβ+ participants with hypertension did not have a higher Aβ burden compared to Aβ+ participants without hypertension (*p* = .141). In the ADNI cohort, Aβ+ participants showed higher WMH volume in the occipital (*p* < .001), parietal (*p* < .001), and deep frontal (*p* = .002) ROIs. Aβ+ and Aβ− groups did not differ in the prevalence of hypertension. Cohort characteristics stratified by Aβ and hypertension status are provided in Tables S3 and S4 for Add‐Tau and ADNI, respectively. Tables S5 and S6 show cohort characteristics for the subsamples with ASL MRI.

**TABLE 1:** Participant characteristics in Add‐Tau and ADNI cohorts.

	Add‐Tau		ADNI	
Characteristic	Aβ−	Aβ+	Aβ−	Aβ+
*N*	102	36	289	234
Age at tau PET visit, years, mean (SD) [range]	69.6 (8.7) [51 to 95]	73.1 (8.3) [58 to 90]	71.9 (7.9) [51 to 92]	74.3 (7.3) [57 to 93]^b^
Female sex, *N* (%)	43 (42.2)	9 (25.0)	149 (51.6)	126 (53.8)
*APOE* ε4 carriers, *N* (%)	15 (14.7)	14 (38.9)^a^	58 (20.1)	121 (51.7)^b^
Education, years mean (SD)	16.0 (2.8)	15.8 (3.3)	16.5 (2.5)	16.6 (2.4)
MCI, *N* (%)	22 (21.6)	14 (38.9)	93 (32.2)	99 (42.3)^b^
MMSE, mean (SD)	29.3 (1.1)	28.7 (1.6)^a^	28.8 (1.5)	28.4 (1.9)
Vascular risk, mean (SD) or *N* (%)				
Hypertension	33 (29.4)	22 (61.1)^a^	127 (43.9)	105 (44.9)
Systolic BP, mmHg	129.3 (16.6)	136.4 (13.6)^a^	132.5 (15.4)	133.6 (18.8)
Diastolic BP, mmHg	77.6 (8.0)	78.7 (8.6)	74.4 (8.6)	73.9 (10.0)
BMI	25.1 (3.6)	25.6 (4.5)	27.8 (4.9)	27.6 (6.4)
Aβ‐PET and tau‐PET, mean (SD)				
Centiloid	2.1 (6.0)	32.2 (24.1)^a^	−1.8 (8.6)	59.0 (40.1)^b^
EC tau‐PET SUVR	1.02 (0.19)	1.03 (0.28)	1.19 (0.28)	1.46 (0.44)^b^
WMH volume, mL, median (IQR)				
Occipital	1.6 (0.8 to 2.3)	1.8 (0.8 to 3.5)	0.3 (0.1 to 0.9)	0.6 (0.2 to 1.4)^b^
Parietal	0.9 (0.3 to 2.4)	1.8 (0.6 to 4.7)	0.8 (0.3 to 2.9)	1.4 (0.6 to 4.9)^b^
Deep frontal	0 (0 to 0.2)	0.1 (0 to 0.4)^a^	0.0 (0 to 0.2)	0.1 (0 to 0.4)^b^
*N* (%) with ASL MRI	56 (54.9)	22 (61.1)	48 (16.1)	41 (17.5)

*Note*: In the Add‐Tau cohort, regional WMH volume was not available for four Aβ− and three Aβ+ participants.

Abbreviations: *APOE*, apolipoprotein E; ASL, arterial spin labeling; BP, blood pressure; EC, entorhinal cortex; HT, hypertension; IQR, interquartile range; MCI, mild cognitive impairment; MMSE, Mini‐Mental State Examination; MRI, magnetic resonance imaging; PETT, positron emission tomography; SD, standard deviation; SUVR, standardized uptake value ratio; WMH, white matter hyperintensity.

^a^ Add‐Tau Aβ− significantly different from Add‐Tau Aβ+.

^b^ ADNI Aβ− significantly different from ADNI Aβ+.

### 3.2 Associations among hypertension, amyloid, and regional WMH burden

In the Add‐Tau cohort, after adjustment for age, sex, and *APOE* ε4 status, Aβ+ participants were more likely to be hypertensive (*p* = .008; Table S7), but no differences in regional rWMH burden were observed (all *p*’s > .256; Table S8). In the ADNI cohort, after adjustment for covariates, Aβ+ and Aβ− participants did not differ in the prevalence of hypertension (*p* = .547). Compared to Aβ− participants, Aβ+ ADNI participants exhibited higher occipital (*p* = .031) and parietal (*p* = .026) but not deep frontal (*p* = .272) rWMH burden after covariate adjustment.

### 3.3 Aβ‐dependent associations between hypertension and WMH burden with EC tau, EC thickness, and memory

**FIGURE 1:**
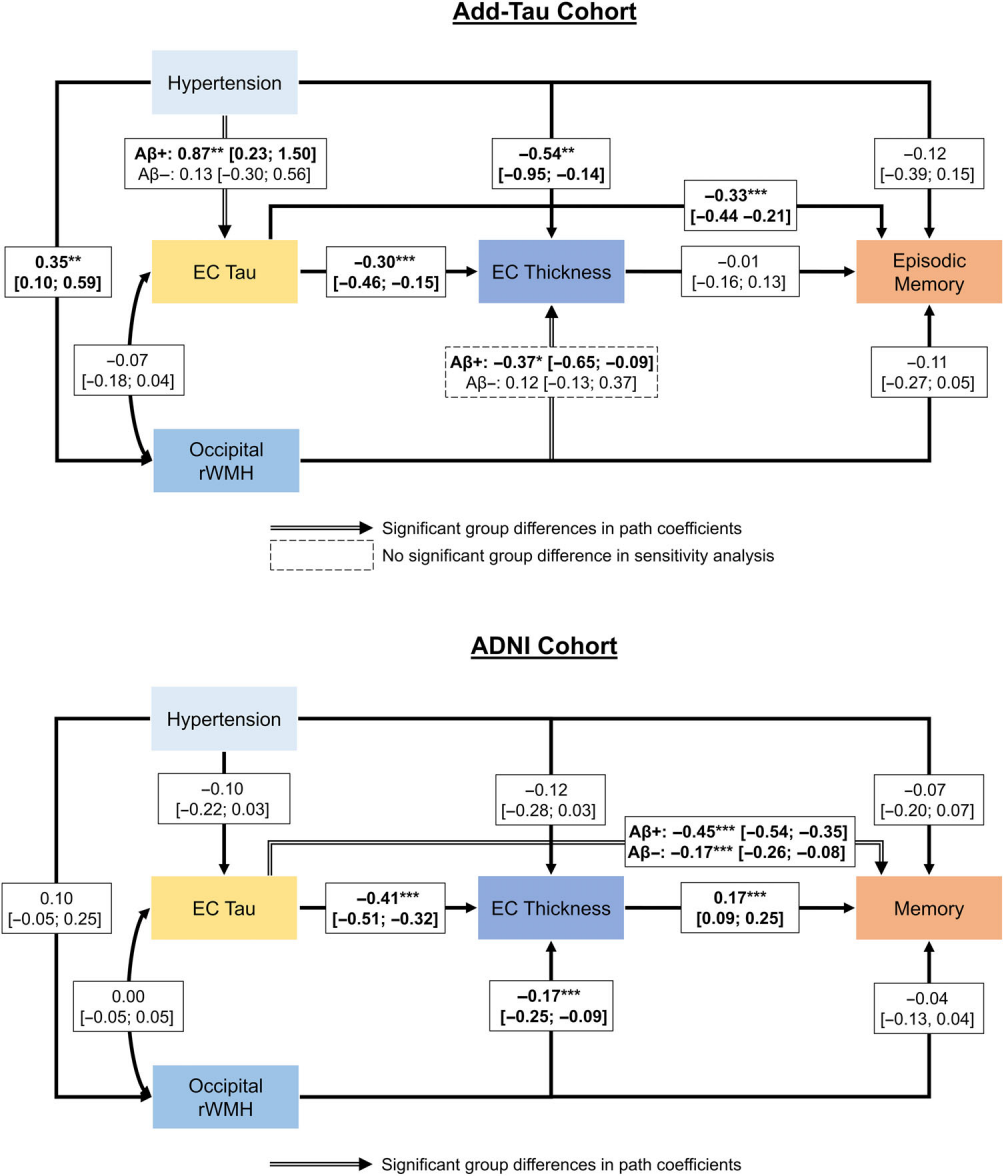
The deleterious effect of hypertension acts through several pathways, some of which differ depending on Aβ exposure. The left‐right‐headed arrow indicates the residual covariance between occipital rWMH burden and EC tau. The values in the boxes indicate path coefficients with the 95% confidence interval in brackets and a significance level of *p < .05, **p < .01, and ***p < .001. Path coefficients were estimated separately for low Aβ (Aβ−) and high Aβ (Aβ+) groups for paths where constraining the coefficients led to a significant reduction in model fit. EC, entorhinal cortex; rWMH, relative white matter hyperintensities.

Figure 1 illustrates the results of the final structural equation models for occipital rWMH burden in the Add‐Tau and ADNI cohorts. Results for all models are summarized in Table 2. In the Add‐Tau cohort, we found significant differences between Aβ+ and Aβ− groups on the paths from hypertension to EC tau (likelihood ratio test, *p_FDR_* = .050, Table S9) and occipital WMHs to EC thickness (*p_FDR_* = .050). Hypertension was associated with higher EC tau pathology in Aβ+ but not Aβ− individuals. Similarly, higher occipital rWMH burden was associated with lower EC thickness in Aβ+ but not Aβ− individuals. Hypertension was also associated with lower EC thickness and increased occipital WMH burden. The interactive association between Aβ burden and hypertension status on EC tau was confirmed in a linear regression model adjusting for age, sex, and APOE ε4 status using a continuous Centiloid variable (β = 0.007, *p *= .002). The interaction was also significant in a linear regression model restricted to Aβ+ individuals (β = 0.007, *p *= .018). In the ADNI cohort, we found a significant difference between Aβ+ and Aβ− individuals on the path from EC tau to memory (*p_FDR_ *< .001, Table S10). While higher EC tau was associated with worse memory performance in both groups, the association was considerably stronger in Aβ+ compared to Aβ− individuals. Notably, and in contrast to the Add‐Tau cohort, we observed a negative association between hypertension and EC tau in the Aβ+ group when no path constraints were applied (β = −0.32, 95% CI: −0.60 to −0.05, *p *= .022). However, since the groups did not differ significantly on this path (*p_FDR_* = .196), it was estimated using the combined sample in the model. In the final model, the path was not significant (Table 2).

**TABLE 2:** Path estimates for rWMH volumes in OCC, PAR, and DFRO from separate structural equation models.

		Add‐Tau			ADNI		
Model	Path	Coefficient [95% CI]	*p*‐value	Group	Coefficient [95% CI]	*p*‐value	Group
All	Hypertension → EC tau	0.13 [−0.30 to 0.56]	.561	Aβ−	−0.10 [−0.22 to 0.03]	.140	Combined
		**0.87 [0.23 to 1.50]**	**.007**	**Aβ+**			
All	Hypertension → EC thickness	**−0.54 [−0.95 to −0.14]**	**.009**	**Combined**	−0.12 [−0.28 to 0.03]	.115	Combined
All	Hypertension → EM	−0.12 [−0.39 to 0.15]	.389	Combined	−0.07 [−0.20 to 0.07]	.328	Combined
All	EC Tau → EC thickness	**−0.30 [−0.46 to −0.15]**	**<.001**	**Combined**	**−0.41 [−0.51 to** −**0.32]**	**<.001**	**Combined**
All	EC tau → EM	**−0.33 [−0.44 to −0.21]**	**<.001**	**Combined**	**−0.17 [−0.26 to −0.08]**	**<.001**	**Aβ−**
					**−0.45 [−0.54 to −0.35]**	**<.001**	**Aβ+**
All	EC thickness → EM	−0.01 [−0.16 to 0.13]	.844	Combined	**0.17 [0.09 to 0.25]**	**<.001**	Combined
OCC	Hypertension → WMH	**0.35 [0.10 to 0.59]**	**.005**	**Combined**	0.10 [−0.05 to 0.25]	.202	Combined
OCC	WMH → EC thickness	0.12 [−0.13 to 0.37]	.349	Aβ−	**−0.17 [−0.25 to −0.09]**	**<.001**	**Combined**
		**−0.37 [−0.65 to −0.09]**	**.010**	**Aβ+**			
OCC	WMH → episodic memory	−0.11 [−0.27 to 0.05]	.175	Combined	−0.04 [−0.13 to 0.04]	.269	Combined
OCC	WMH ↔ EC tau	−0.07 [−0.18 to 0.04]	.185	Combined	0.0 [−0.05 to 0.05]	.965	Combined
PAR	Hypertension → WMH	0.12 [−0.15 to 0.39]	.377	Combined	**0.15 [0.02 to 0.29]**	**.030**	**Combined**
PAR	WMH → EC thickness	−0.08 [−0.28 to 0.12]	.415	Combined	**−0.16 [−0.24 to −0.09]**	**<.001**	**Combined**
PAR	WMH → episodic memory	0.09 [−0.07 to 0.26]	.275	Combined	−0.09 [−0.18 to 0.01]	.070	Aβ−
					**−0.27 [−0.40 to −0.13]**	**<.001**	**Aβ+**
PAR	WMH ↔ EC tau	0.02 [−0.06 to 0.09]	.649	Combined	0.02 [−0.02 to 0.07]	.319	Combined
DFRO	Hypertension → WMH	**0.33 [0.02 to 0.64]**	**.035**	**Combined**	**0.27 [0.13 to 0.42]**	**<.001**	**Combined**
DFRO	WMH → EC thickness	−0.14 [−0.29 to 0.02]	.079	Combined	**−0.17 [−0.25 to −0.08]**	**<.001**	**Combined**
DFRO	WMH → episodic memory	0.07 [−0.04 to 0.18]	.211	Combined	**−0.13 [−0.21 to −0.06]**	**.001**	**Combined**
DFRO	WMH ↔ EC tau	0.03 [−0.08 to 0.13]	.616	Combined	0.04 [−0.01 to 0.09]	.132	Combined

*Note*: The *Model* column specifies the source model for the estimates. For paths not involving rWMH volume, “All” is used. The estimates reported in the table for these paths were derived from the occipital model, but they were nearly identical across all models. The *Group* column indicates whether the path has been estimated separately for Aβ− and Aβ+ groups (indicating significant group differences with FDR‐corrected *p* ≤ .05) or using the combined sample (indicating no significant group differences).

Abbreviations: ADNI, Alzheimer's Disease Neuroimaging Initiative; EC, entorhinal cortex; DFRO, deep frontal ROI; FDR, false discovery rate; OCC, occipital; PAR, parietal; ROI, regions of interest; rWMH, relative WMH; WMH, white matter hyperintensities.

Significant paths (*p* < .05) are highlighted in bold.

We repeated the analysis for rWMH volume in parietal and deep frontal ROIs (Table 2, Figures S5 and S6). The only significant difference between Aβ+ and Aβ− groups was found in the ADNI cohort on the path from parietal rWMH to memory performance. Specifically, higher parietal rWMH was associated with worse memory performance in Aβ+ but not Aβ− individuals. Higher deep frontal rWMH burden was associated with worse memory performance as well, and both higher parietal and deep frontal rWMH burdens were associated with decreased EC thickness in the ADNI cohort. In both cohorts, hypertension was associated with rWMH burden in the deep frontal region.

Group differences on certain paths (or the absence thereof) may be influenced by effects of covariates that differ between Aβ+ and Aβ− individuals. To address this, we repeated the analysis, restricting the effects of covariates to be the same across both groups (Tables S9 and S10). In this more restricted model, we did not observe a significant difference between Aβ+ and Aβ− individuals on the path from occipital rWMH to EC thickness in the Add‐Tau cohort, resulting in a non‐significant path (β = −0.02, 95% CI: −0.22 to 0.17, *p *= .805). The results in the ADNI cohort remained unchanged.

We identified five outliers in the Add‐Tau cohort and 21 outliers in the ADNI cohort based on Mahalanobis distance. In the Add‐Tau cohort, all outliers were individuals diagnosed with MCI, with four of them exhibiting a high Aβ burden (Centiloid > 71). The results depicted in Figure 1 and reported in Table 2 remained largely unchanged after outlier removal. In the ADNI cohort, outliers were predominantly participants with a large time difference between Aβ and tau‐PET scans, including 16 out of the 17 participants with a time difference exceeding 6 months. After excluding these outliers, the significance of the association between EC tau and memory performance in Aβ− individuals was greatly reduced (*p* = .048). Other findings remained largely unchanged. Results did not differ when individuals scanned on a Philips scanner were excluded from the analysis (Table S11 and Figure S7).

### 3.4 Influence of hypertension and Aβ burden on CBF, WMH, and EC tau

We next analyzed participants from the Add‐Tau (*n* = 78) and ADNI (*n* = 89) cohorts with available ASL data to gain insight into the potential role of CBF in the established associations between Aβ and hypertension with both rWMHs and EC tau. Since we did not detect an interaction between Aβ burden and hypertension on regional rWMH in our previous analyses, we hypothesized that if a potential influence of hypertension and Aβ on regional WMH burden was mediated through altered CBF, these effects would operate independently. In contrast, given our observation of an interactive association between Aβ burden and hypertension on EC tau in the Add‐Tau cohort, we hypothesized that if this effect was mediated through altered CBF, it would vary depending on the presence or absence of Aβ. Consequently, we formulated two distinct models to investigate these hypotheses within the Add‐Tau cohort, characterized by low Aβ pathology, and the ADNI cohort, representing more advanced disease stages with high Aβ pathology. For the Add‐Tau cohort, we report the results using non‐PVC CBF values in Figure S8.

#### 3.4.1 CBF in relationships among hypertension, Aβ burden, and regional WMH burden

**FIGURE 2:**
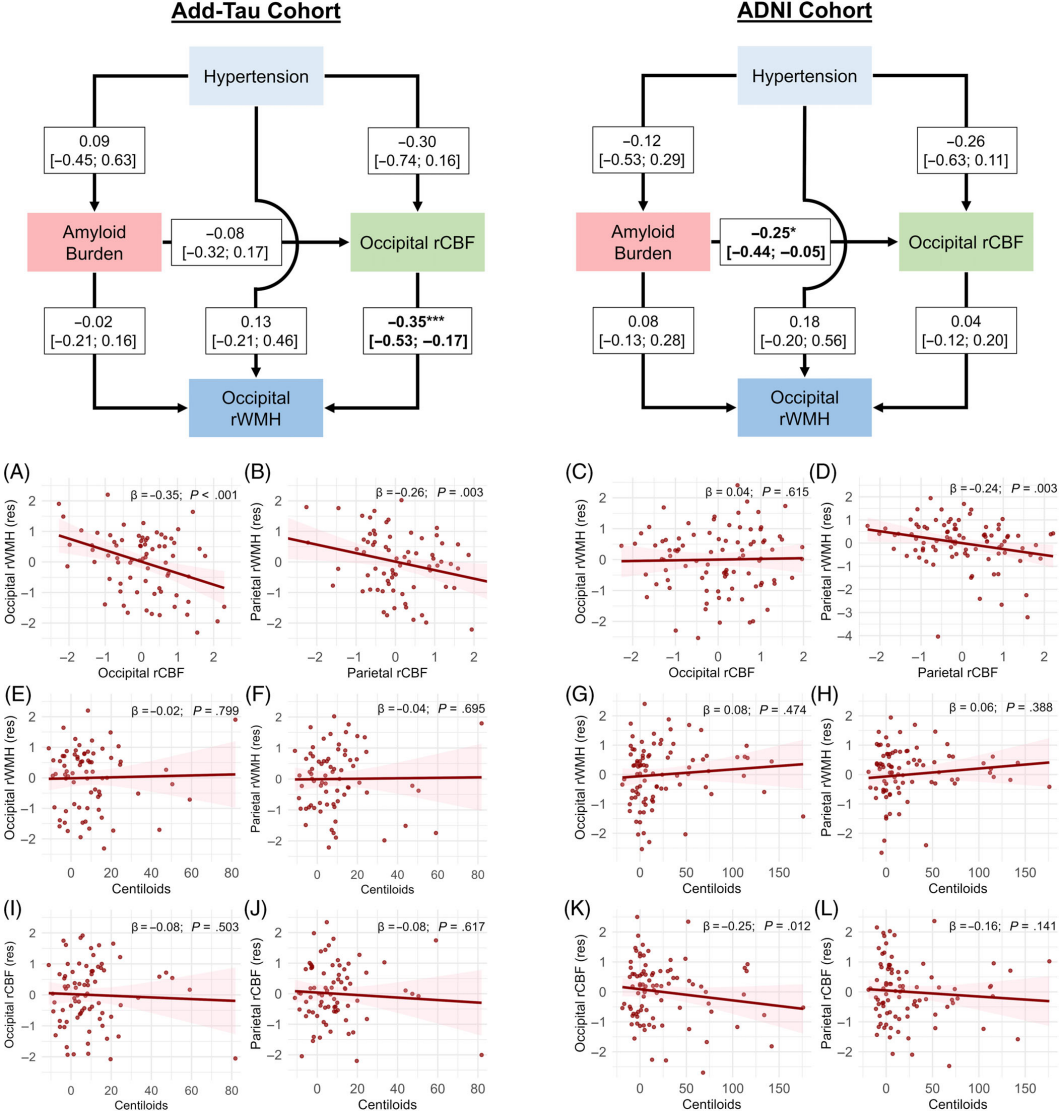
Relationships among hypertension, continuous Aβ burden, regional rCBF, and regional rWMH volume in Add‐Tau and ADNI cohorts. The models and scatterplots on the left‐hand side illustrate associations for the Add‐Tau cohort, while those on the right side illustrate associations for the ADNI cohort. The panels present scatterplots depicting the associations between regional rWMH and regional rCBF (A–D), Aβ burden and regional rWMH (E–H), and Aβ burden and regional rCBF (I–L). The values in the boxes in the structural equation models indicate path coefficients with the 95% confidence interval in brackets and significance levels of *p < .05 and ***p < .001. β coefficients and *p*‐value displayed in the scatterplots are extracted from the respective paths within the corresponding structural equation model. The values on the *y*‐axis in the plots represent residuals obtained after regressing the influences of age and sex. ADNI, Alzheimer's Disease Neuroimaging Initiative; rCBF, relative cerebral blood flow; rWMH, relative white matter hyperintensities.

We first investigated whether Aβ, rCBF, and hypertension were associated with regional rWMH. The results for occipital and parietal ROIs are summarized in Figure 2. In the Add‐Tau cohort, the presence of hypertension was associated with lower frontal rCBF (β = −0.48, 95% CI: −0.93 to −0.04, *p *= .034) but was not significantly associated with continuous Aβ burden, regional rWMH, or rCBF in the occipital or parietal ROIs. Aβ burden showed no association with either regional rCBF or regional rWMH volume. However, detecting such associations was unlikely given the low Aβ burden observed in this sample. In the ADNI cohort, higher Aβ burden was associated with reduced rCBF in the occipital region, but not in the frontal or parietal regions or with regional rWMH exposure. Higher parietal rCBF was associated with lower parietal rWMH burden in both cohorts. The same path was significant for the occipital lobe in the Add‐Tau cohort, but not in the ADNI cohort. The removal of outliers and adjustment for potential confounders did not alter these findings.

#### 3.4.2 Synergistic association of MTL rCBF and Aβ burden on EC tau

**FIGURE 3:**
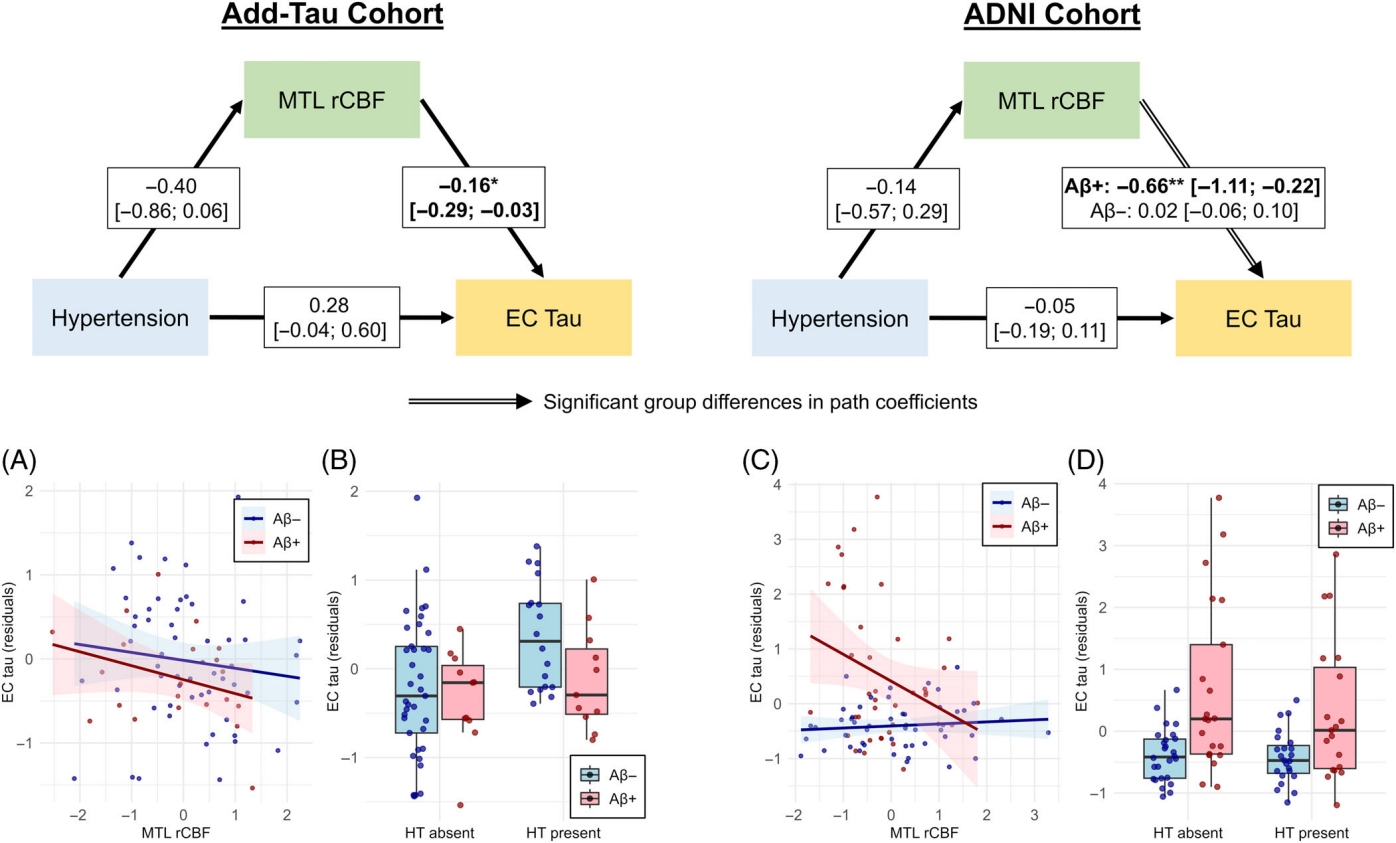
Mediating role of MTL rCBF in association between hypertension and EC tau depending on Aβ status in Add‐Tau and ADNI cohorts. The models and plots on the left‐hand side illustrate associations for Add‐Tau cohort, while those on the right‐hand side illustrate associations for ADNI cohort. In the Add‐Tau cohort, one outlier was excluded from the plots but was included in the statistical analysis. The values in the boxes in the moderated‐mediation models indicate path coefficients with the 95% confidence interval in brackets and significance levels of *p < .05 and **p < .01. EC tau values in the plots represent residuals obtained after regressing the effects of age and sex. ADNI, Alzheimer's Disease Neuroimaging Initiative; EC, entorhinal cortex; MTL, medial temporal lobe; rCBF, relative cerebral blood flow.

**FIGURE 4:**
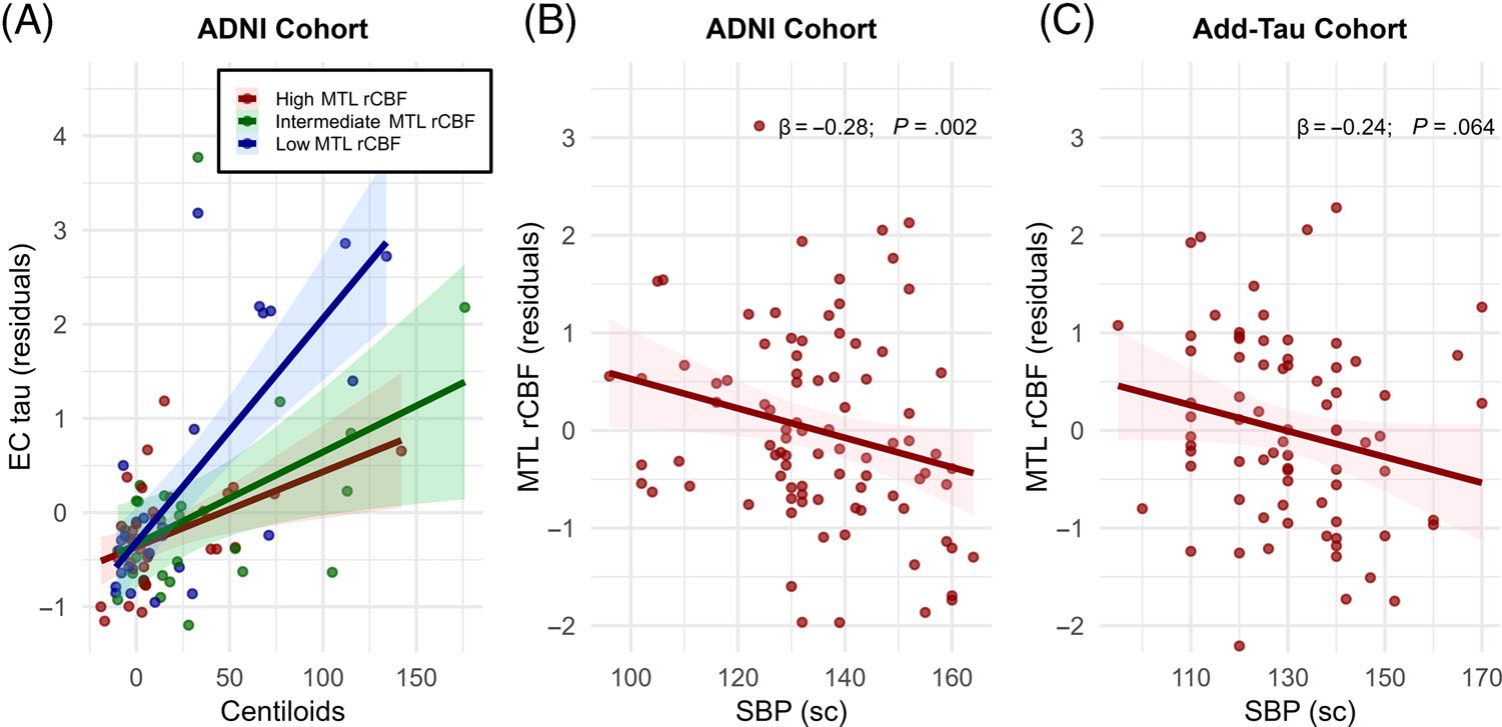
MTL rCBF modifies the association between Aβ burden and EC tau in the ADNI cohort (A) and is lower in individuals with high SBP at sc (B, C). In *A*, tertiles of MTL rCBF divided ADNI participants into high, intermediate, and low groups. In *B* and *C*, *β* coefficients and *p*‐values displayed are extracted from the respective paths within the corresponding structural equation model. The values on the *y*‐axis in the plots represent residuals obtained after regressing the influences of age and sex. ADNI, Alzheimer's Disease Neuroimaging Initiative; MTL, medial temporal lobe; rCBF, relative cerebral blood flow; sc, screening visits.

Next, we investigated whether Aβ status modified a potentially CBF‐mediated association between hypertension and EC tau (Figure 3). In both cohorts, we found no differences between Aβ+ and Aβ− groups on the paths from hypertension to EC tau and hypertension to MTL rCBF (Tables S12 and S13). The association between MTL rCBF and EC tau was significantly different between Aβ+ and Aβ− groups in the ADNI cohort (*p_FDR_* = .017) but not in the Add‐Tau cohort (*p_FDR_* = .987). Specifically, in the ADNI cohort, higher MTL rCBF was associated with lower EC tau in Aβ+ but not Aβ− individuals. This interaction between Aβ burden and MTL rCBF on EC tau is further illustrated in Figure 4A. Similar to the ADNI cohort, the Add‐Tau cohort also showed a significant negative association between MTL rCBF and EC tau in Aβ+ but not in Aβ− individuals (Aβ+: β = −0.18, 95% CI: −0.33 to −0.02, *p* = .031; Aβ−: β = −0.10, 95% CI: −0.35 to 0.16, *p* = .466). However, since the groups did not differ significantly on this path, it was set to be equal in the final model.

The results for the ADNI cohort were consistent across all sensitivity analyses. In the Add‐Tau cohort, the significance of the MTL rCBF to EC tau pathway diminished after adjusting for MMSE (β = ‐0.14, *p* = 0.052). Additionally, following adjustment for BMI (β = 0.29, *p* = .040) or removing one influential individual (β = 0.45, *p* = .006), all resulted in a significant direct association between hypertension and EC tau.

### 3.5 SBP at screening visits

We repeated the analyses using SBP measurements instead of a hypertension diagnosis. In the Add‐Tau and ADNI cohorts, SBP was assessed with a median (IQR) of 1.5 (1 to 4.1) years and 1 (0 to 6) years before the ASL acquisition, respectively. In our first model (compare Figure 1 and Figure S9), unlike a hypertension diagnosis, SBP was not associated with EC tau, EC thickness, or regional rWMH burden in the Add‐Tau cohort. Similarly, in the ADNI cohort, SBP was not related to EC tau, EC thickness, or regional rWMH burden. In the second model (compare Figure 2 and Figure S10), we observed results comparable to those obtained when using a hypertension diagnosis. Specifically, higher SBP was associated with lower frontal rCBF (β = −0.29, p = .010) in the Add‐Tau cohort, but no significant associations were observed in the ADNI cohort. Additionally, in line with the observed association between hypertension and higher Aβ burden in the full Add‐Tau cohort, SBP was associated with increased continuous Aβ burden (β  = 0.21, *p* = .013). In the third model (compare Figure 3 and Figure S11), akin to the findings with hypertension diagnosis, higher SBP was marginally associated with lower MTL rCBF in the Add‐Tau cohort (β = −0.24, *p* = 0.064). In the ADNI cohort, compared to hypertension which showed no association with MTL rCBF, higher SBP was significantly associated with lower rCBF in the MTL (β = −0.25, *p* = .003). The relationship between SBP and MTL rCBF is depicted in Figure 4B for the Add‐Tau cohort and in Figure 4C for the ADNI cohort.

## 4 DISCUSSION

By analyzing two independent samples – one with a generally low Aβ burden (Add‐Tau cohort) and another in more advanced disease stages with a high Aβ burden (ADNI cohort) – our study highlights that hypertension adversely affects brain health through both AD pathology‐dependent and AD pathology‐independent pathways. Additionally, our study provides evidence that both Aβ and hypertension may be risk factors contributing to increased WMH burden and EC tau. Particularly in posterior brain regions, Aβ and hypertension may act partially through a shared pathological mechanism, manifested as impaired cerebral perfusion.

Hypertension is a major risk factor for increased WMH volume^7^ and MTL atrophy^42^ that has also been found to be associated with tau pathology independently and interactively with brain amyloidosis.^2^, ^43^, ^44^ Consistent with these studies, in the Add‐Tau cohort, hypertensive individuals were more likely to be Aβ+, exhibited lower EC thickness, and increased EC tau, the latter particularly in the presence of elevated Aβ burden. Importantly, our results indicate that several concurrent mechanisms may operate and that some of these mechanisms are contingent upon the presence of Aβ pathology. Specifically, occipital WMH burden was related to lower EC thickness in the presence of Aβ, and hypertension interacted with Aβ to predict higher EC tau. Elevated EC tau burden, in turn, was closely associated with reduced EC thickness and poorer episodic memory performance.^24^ The interaction was observed using a low Aβ positivity threshold of 12 Centiloids, aligning with a prior study that demonstrated a significant interactive association between Aβ and the Framingham Heart Study cardiovascular disease risk score on tau accumulation at 11.8 Centiloids.^2^ In the ADNI cohort, hypertension was less strongly associated with typical AD pathology, and the association between hypertension on EC thickness was primarily mediated through higher WMH burden. Notably, even after accounting for the effects of EC tau and EC thickness, parietal WMH burden was associated with worse memory performance in the presence of Aβ, while in the deep frontal region, this association was independent of Aβ. These findings underscore the varied mechanisms by which hypertension could contribute to the development of cognitive impairment and emphasize the interconnectedness of vascular and AD pathophysiological processes.^1^, ^45^

Our study identified notable differences between the cohorts in terms of associations with hypertension (summarized in Table S14). In the ADNI cohort, the adverse effects of hypertension were particularly related to WMH burden, whereas in the Add‐Tau cohort, associations were found between hypertension and AD pathology, EC thickness, and WMH burden. Notably, in ADNI Aβ+ individuals, hypertension was associated with lower EC tau, contrary to our observation in the Add‐Tau cohort and to our expectation based on previous studies.^2^, ^46^, ^47^ These findings may reflect cohort differences related to disease stage and recruitment strategies. The ADNI cohort includes participants recruited to represent individuals typical of AD clinical trials who are characterized by low vascular risk (Modified Hachinski Ischemic Score ≤ 4) and, in cases of abnormal cognition, present primarily memory impairment.^48^ Consequently, many participants exhibit significant Aβ pathology and are carriers of an APOE ε4 allele. Given the adverse effect of vascular pathologies on cognitive performance observed particularly in the presence of Aβ and in APOE ε4 carriers,^49^, ^50^, ^51^ those individuals recruited with a high vascular pathological burden might tend to be those with lower tau pathology. The Add‐Tau cohort also includes participants with relatively low vascular risk, but, in contrast to the ADNI cohort, it includes fewer APOE ε4 carriers, and most Aβ+ individuals still show a relatively low Aβ burden. Consequently, the Add‐Tau cohort may be more representative of a healthy older cohort in which the underlying pathological burden is low but has more diverse etiologies.

While a direct relationship between posterior white matter lesions and tau pathology may exist in patients with severe AD,^52^ our results suggest that in cognitively normal and MCI individuals, hypertension and Aβ pathology may serve as shared risk factors for WMHs and EC tau. Brain hypoperfusion may be one common upstream candidate that mediates these associations,^1^, ^19^, ^20^, ^53^ and our results provide some support for this possibility. We observed that a region‐specific decrease in gray matter CBF correlated with increased WMH volume, and we found that lower MTL CBF was associated with increased EC tau in Aβ+ individuals. Although the association between EC tau and MTL CBF in the Add‐Tau cohort appeared to be independent of Aβ status, we note that it was driven by Aβ+ individuals. Due to the limited number Aβ+ individuals with substantially elevated Aβ burden in this cohort, we likely did not have enough power to detect a statistical difference between Aβ− and Aβ+ groups. The largely absent association between hypertension and regional CBF may be attributed to cerebrovascular autoregulation, which helps maintain stable CBF across a wide range of mean arterial pressures.^54^ Interestingly, we found that higher SBP assessed at screening visits was a better predictor of lower CBF than a hypertension diagnosis. The specificity of this observation for the MTL aligns with recent research highlighting the MTL's selective vulnerability to blood pressure fluctuations.^55^

Our findings also offer support for the possibility that the link between Aβ burden and posterior WMH volume might be mediated by CBF. This interpretation is based on the combined results of the two cohorts, given that occipital WMH volume, which was clearly correlated with occipital CBF in the Add‐Tau cohort, showed no discernible relationship in the ADNI cohort. The strength of the Aβ‐to‐CBF associations followed a posterior‐to‐anterior gradient, being strongest for the occipital lobe and weakest for the frontal lobe. One explanation is that Aβ‐PET burden may function as a proxy for an individual's underlying cerebral amyloid angiopathy (CAA),^56^, ^57^ which probably also progresses in a posterior‐to‐anterior fashion.^18^ In individuals with CAA, WMH burden in posterior brain regions is increased^58^ and vasodilation to physiological stimuli in the occipital lobe is reduced.^59^ Moreover, in line with our interpretation of shared mechanisms contributing to tau pathology and WMH burden, a recent study found that the association between global WMH volume and temporal tau‐PET burden was no longer significant after accounting for cerebral microbleeds, a marker for CAA.^46^ Alternatively, Aβ oligomers have also been found to generate pericyte constriction, potentially resulting in decreased CBF and compromised energy supply, but such changes are likely to be too subtle to be measured by ASL.^15^ Our finding of a relationship between Aβ and CBF contrasts with a previous study that did not find such an association.^60^ Decreased CBF may mirror the functional deterioration of certain brain regions and, particularly in patients with late MCI and AD,^33^ it could be more closely linked to brain atrophy. However, our analysis did not include participants with AD and the occipital lobe is typically neither the primary site of Aβ accumulation nor atrophy until more advanced disease stages.^61^ The association also remained significant when controlling for our measure of disease stage (MMSE score). Nevertheless, we cannot exclude the possibility that decreased CBF in some regions may be due to reduced metabolic demands possibly related to brain dysfunction which, especially in MTL regions, could provide a plausible explanation for the association with EC tau.

The use of multiple modalities in this study warrants important methodological considerations. Methodological and technical aspects may interact across modalities within a given region, creating the appearance of an association. For instance, WMHs, which typically show reduced CBF,^62^ may have led to lower gray matter CBF due to erroneous image registration and partial volume contamination. We attempted to mitigate the influence of such methodological aspects to the best of our ability by inspecting image co‐registration and manually correcting WMH and ROI delineations, but we may not have been able to completely eliminate these effects. Another consideration pertains to the causality of the factors under investigation. Although our analyses and discussions frequently imply causality, it is important to recognize that these associations could be independent events or be subject to reverse causality. For instance, some longitudinal studies indicate that baseline WMH burden predicts changes in gray matter CBF, but baseline CBF does not predict WMH progression.^63^, ^64^ Some of the studied events may also be indirectly related through other vascular‐physiological properties. For example, associations between gray matter CBF and WMHs or EC thickness and WMHs could be confounded by increased arterial transit time^65^ or arterial stiffness, respectively.^66^

There are other limitations to this study. Due to the cross‐sectional design, further studies are required to determine whether the identified correlations can be confirmed prospectively. Inclusion criteria employed for the Add‐Tau and ADNI cohorts exclude individuals diagnosed with certain cardiovascular conditions and participants in both cohorts are generally well educated. Thus, both cohorts are likely underrepresenting individuals with high vascular risk, and the effects of hypertension may be underestimated. Additionally, the sample size in the subsamples with ASL MRI was small, and direct comparison between the two cohorts was complicated by the different disease stages represented. Our analysis did not account for the time of hypertension diagnosis, which may have impacted our findings as hypertension in midlife is associated with worse cognitive outcomes.^17^ Finally, due to limited subgroup sample sizes, the present study did not explore moderating effects of the APOE ε4 allele and sex, which could be important modifiers for certain paths.^17^, ^37^

In summary, our results suggest that hypertension has a multifaceted influence in the development of AD dementia and demonstrate the interconnectedness of vascular and AD pathophysiological processes. Individuals with hypertension exhibited increased AD pathology and a higher WMH burden, particularly in the frontal lobe. Both pathologies were independently associated with reduced EC thickness and worse memory performance. Furthermore, regional cerebral perfusion reductions, potentially downstream of Aβ and hypertension, were associated with WMH burden in the posterior brain and, interactively with Aβ, increased EC tau. Longitudinal studies will be crucial to help establish the temporal sequence of these factors and determine whether controlling blood pressure also reduces AD pathology.

## CONFLICT OF INTEREST STATEMENT

Christoph Hock and Roger M. Nitsch are employees and shareholders of Neurimmune AG, Switzerland. Dario Bachmann, Antje Saake, Sandro Studer, Andreas Buchmann, Katrin Rauen, Esmeralda Gruber, Lars Michels, Anton Gietl, and Valerie Treyer declare no relevant conflicts of interest. Author disclosures are available in the supporting information.

## CONSENT STATEMENT

All participants provided written informed consent.

## Supporting information

Supporting Information

Supporting Information

## Data Availability

For data related to the Add‐Tau cohort, the raw data supporting the conclusions of this article will be made available by the authors upon reasonable request after evaluation by the authors and, if applicable, by the local ethics authority. ADNI data are publicly available on the ADNI database (http://adni.loni.usc.edu) upon registration and compliance with the data usage agreement.

## ADNI COLLABORATORS

### 1

**Table:**

Name	Location	Role	Contribution
Michael W. Weiner, MD	University of California, San Francisco	Principal investigator	PI
John Q. Trojanowski, MD, PhD	University of Pennsylvania	Core leader	Coordinated biomarker core
Leslie Shaw, PhD	University of Pennsylvania	Core leader	Coordinated biomarker core
Laurel Beckett, PhD	University of California, Davis	Core leader	Coordinated biostatistics core
Paul Aisen, MD	University of Southern California	Core leader	Coordinated clinical core
Ronald Petersen MD, PhD	Mayo Clinic	Core leader	Coordinated clinical core
John C. Morris, MD	Washington University St. Louis	Core leader	Coordinated neuropathology core
Richard J. Perrin, MD, PhD	Washington University St. Louis	Core leader	Coordinated neuropathology core
Arthur W. Toga, PhD	University of Southern California	Core leader	Coordinated informatics core
Clifford Jack, MD	Mayo Clinic, Rochester, Minnesota	Core leader	Coordinated MRI core
Robert C. Green, MD, MPH	Brigham and Women's Hospital/Harvard Medical School	Chair	Data and publications committee
William Jagust, MD	University of California, Berkeley	Core leader	Coordinated PET core
Andrew J. Saykin, PsyD	Indiana University	Core leader	Coordinated genetics core

*Note*: Part of the data used in the preparation of this article was obtained from the Alzheimer's Disease Neuroimaging Initiative (ADNI) database (adni.loni.usc.edu). As such, the investigators within the ADNI contributed to the design and implementation of ADNI and/or provided data but did not participate in the analysis or writing of this report. A complete listing of ADNI investigators can be found at: http://adni.loni.usc.edu/wp‐content/uploads/how_to_apply/ADNI_Acknowledgement_List.pdf.
