# Supplementary material for: Hypertension and cerebral blood flow in the development of Alzheimer's disease
Source: Alzheimers Dement. 2024 Sep 10;20(11):7729–44. doi: 10.1002/alz.14233 (PMC11567827; doi:10.1002/alz.14233)
Supplement: Supplementary file 1 — Supporting Information [file ALZ-20-7729-s002.docx]

**Hypertension and Cerebral Blood Flow in the Development of Alzheimer’s Disease**

– Supplementary Materials –

Dario Bachmann, Antje Saake, Sandro Studer, Andreas Buchmann, Katrin Rauen, Esmeralda Gruber, Lars Michels, Roger M Nitsch, Christoph Hock, Anton Gietl, and Valerie Treyer for the Alzheimer’s Disease Neuroimaging Initiative

**Supplementary Methods**

**Cerebral Blood Flow Calculation.** FMRIB Software Library^1^ (FSL) was used for post-processing of ASL images. The perfusion-weighted (PW) images as directly provided by the GE scanner were used. These images did not incorporate motion correction. PW images were co-registered to each participant’s FreeSurfer-processed and bias field corrected T1-weighted image using a boundary-based cost function,^2^ which was reviewed for accuracy. PW images were divided by GE scanners global scaling factor of 32, and then number of excitations (NEX) 3. PW images were corrected for partial volume effects in native ASL space using a linear regression algorithm^3^ using the BASIL toolbox included within FSL.^4^ The asl_file command line tool was used for the PVC with a regression-kernel sizes of 5 × 5 × 1. The gray matter and white matter probability maps needed for this procedure were segmented from the T1 image (using fsl_anat). Probability maps were registered to ASL space using the inverse transformation derived from the registration of ASL data to structural image (using applywarp). This involved transforming the probability maps on a four times super-sampled resolution of the ASL data with spline interpolation. Subsequently, probability maps in the low-resolution ASL image space were derived by integrating over the relevant regions.^5, 6^ The M0 proton-density-weighted reference images were obtained with a saturation recovery acquisition using readout parameters identical to the ASL readout. These maps were converted to M_0a_ by the following equation:

$$M_{0a}= \frac{PD}{\lambda_{GM}\cdot(1-e^{\frac{-TR}{{T1}_{Tissue}}})}$$

where TR is the saturation recovery time (2 s), T1_Tissue_ is the relaxation time of gray matter tissue (1.2 s at 3T), and λ_GM_ is the gray matter brain-blood water partition coefficient (0.9 ml/g).^7^ After masking the M_0a_ images using the FreeSurfer-generated brain mask, the images where smoothed and CBF maps were calibrated on a voxel-wise basis using a single compartment quantification model^7^:

$$CBF= \frac{6000\cdot{PW}_{PVC}\cdot e^{PLD/T_{1, blood}}}{2\cdot\varepsilon\cdot M_{0a}\cdot T_{1,blood}\cdot(1-e^{-\frac{-\tau}{T_{1, blood}}})} [ml/100g/min]$$

where PW_PVC_ represents the PVC difference images between control and label and M_0a_ the equilibrium magnetization of arterial blood. PLD is the post-labeling delay (2.025 s), T_1,blood_ is the longitudinal relaxation time of arterial blood (1.650 s), and 𝜏 is the labeling duration (1.45 s). ε is the labeling efficiency that is a combination of both the inversion efficiency (0.8) and background suppression efficiency (0.75) resulting in an overall efficiency of 0.6.

CBF maps were then transformed to the FreeSurfer space to compute regional gray matter CBF in the occipital, parietal, frontal, medial temporal region as well as the brainstem which we created by combining relevant Freesurfer-segmented ROIs. The MTL region includes EC, parahippocampal cortex, hippocampus, and amygdala ROIs. Mapping of individual Desikan-Killiany ROIs to the occipital, parietal, and frontal lobes have been described in a previous publication.^8^ However, for both the Add-Tau and ADNI cohorts, we excluded medial and lateral orbitofrontal ROIs because in some participants these regions contained artifactual hyperintense voxels.^9^

**Performance assessment of the Lesion segmentation toolbox (LST) for ADNI participants.** To calculate regional WMH volumes, we used the lesion prediction algorithm that we had also used for WMH determination in the Add-Tau cohort. The lesion prediction algorithm is implemented in the LST toolbox version (www.statistical-modelling.de/lst.html) for SPM. For each scanner manufacturer, we determined the optimal threshold to apply to the lesion probability maps by testing different thresholds on 20 randomly selected subjects. For each individual, we visually inspected the resulting binarized lesion masks for accuracy. For FLAIR scans acquired on SIEMENS or Philips scanners, we applied a threshold of 0.35 to the lesion probability maps. For FLAIR scans acquired on GE scanners, we used a threshold of 0.50. In cases where the scanner-specific threshold performed poorly, an individual threshold was determined. This adjustment was necessary for 46 out of 523 individuals, typically requiring an increase to above 0.90.

In a linear regression model adjusted for age and sex, we observed that the total WMH volume was significantly higher for participants scanned on Philips scanners compared to those scanned on GE (β = 0.708, P < 0.001) or SIEMENS (β = 0.528, P < 0.001). There was no significant difference in WMH volume between SIEMENS and GE (β = 0.15, P = 0.09). To ensure that these results were not driven by differences in the performance of the LST between scanner manufacturers, we first compared our total WMH volumes to the total WMH volumes available from the ADNI website, which are provided by the ADNI MR Core and segmented at the University of California at Davis (UCD). As Supplementary Figure 1 shows, the WMH volumes calculated in the present study using the LST align well with those provided by UCD. When examining UCD WMH volumes, we similarly observed higher WMH volumes for individuals scanned on a Philips scanner compared to GE (β = 0.410, P < 0.001) or SIEMENS (β = 0.448, P < 0.001). Additionally, there was no significant difference between SIEMENS and GE (β = 0.041, P = 0.667). To further ensure that our results are not influenced by systemic WMH volume differences among manufacturers, we conducted a sensitivity analysis by excluding the 70 individuals whose FLAIR images were acquired on a Philips scanner. We repeated the initial analyses, including only individuals scanned on SIEMENS or GE scanners. This analysis yielded virtually the same results as when all participants were included (Figure SMethods).

***Figure SMethods
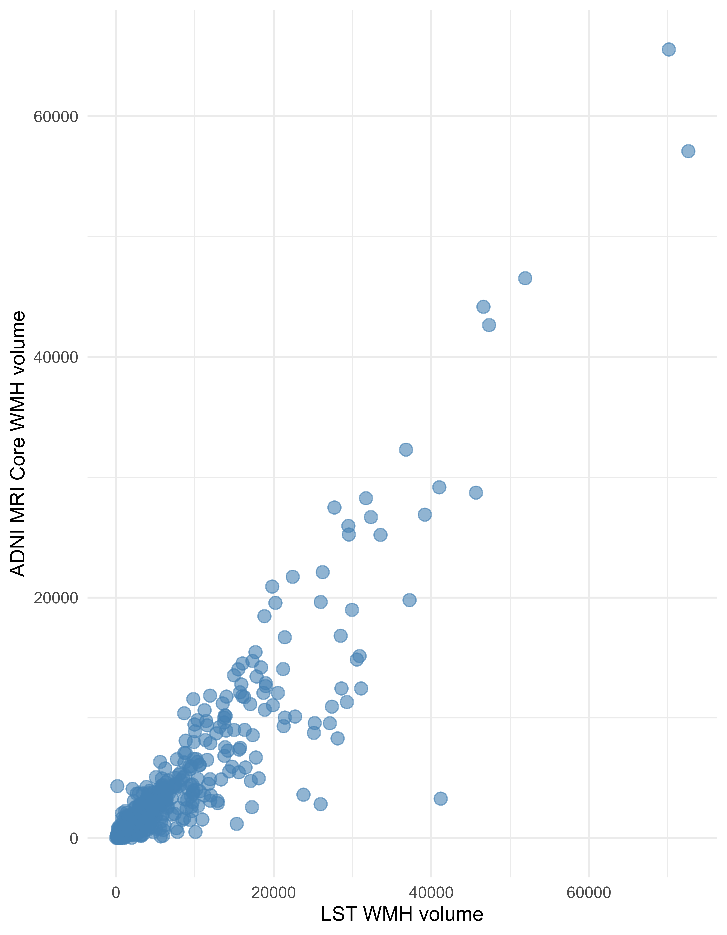
.*** *WMH volumes provided by the ADNI MRI Core show a strong correlation with the WMH outlined in the present study using the LST.*

Together, this suggests that the scanner manufacturer is unlikely to have significantly influenced our results reported in the main analysis. The observed differences might partly be due to the distribution of MCI participants across scanner manufacturers. For Philips, 55% of the participants were diagnoses as MCI, but only 37% and 32% for GE and SIEMENS, respectively.

**Table S1. Absolute mean (SD) regional CBF values in ml/100g/min.** For the Add-Tau cohort, values are provided before and after PVC. For the ADNI cohort, only PVC values were available. Note that the methods used to calculate CBF values and perform PVC differed between the two cohorts. Frontal CBF measurement was missing for one participant in the Add-Tau cohort.

|  | **Add-Tau** | | **ADNI** |
| --- | --- | --- | --- |
| **Region** | **Non-PVC** | **PVC** | **PVC** |
| Occipital | 35.7 (10.1) | 50.0 (13.9) | 39.9 (11.4) |
| Parietal | 39.0 (9.8) | 56.0 (14.2) | 39.1 (10.1) |
| Frontal | 42.8 (8.2) | 61.0 (12.8) | 35.0 (7.8) |
| Medial-temporal | 37.3 (7.1) | 45.7 (9.1) | 35.4 (6.1) |
| Brainstem | 35.3 (7.1) | 51.2 (9.1) | 33.2 (6.4) |

**Table S2. Indices of Fit for Structural Equation Models.** The *Model* column refers to the figures in the main manuscript that illustrate the models corresponding to the fit indices. A good model fit is indicated by non-significant χ^2^ statistics (p > 0.05), SRMR < 0.08 and CFI > 0.95.^10^ However, note that the model fit depends on the sample size, model complexity, strength of regressive paths, and number of variables.^11-13^

| **Model** | **Description** | **χ^2^** | **df** | ***P* value** | **SRMR** | **CFI** |
| --- | --- | --- | --- | --- | --- | --- |
| **Figure 1** | Add-Tau cohort, final model, no equality constraints on covariates | 31.752 | 28 | 0.2847 | 0.034 | 0.98 |
| **Figure 1** | Add-Tau cohort, final model, equality constraints on covariates | 58.715 | 44 | 0.068 | 0.065 | 0.94 |
| **Figure 1** | ADNI cohort, final model, no equality constraints on covariates | 37.285 | 21 | 0.016 | 0.032 | 0.970 |
| **Figure 1** | ADNI cohort, final model, equality constraints on covariates | 51.221 | 35 | 0.038 | 0.041 | 0.971 |
| **Figure 2** | Add-Tau cohort | 6.163 | 3 | 0.104 | 0.042 | 0.964 |
| **Figure 2** | ADNI cohort | 1.794 | 3 | 0.616 | 0.022 | 1.00 |
| **Figure 3** | Add-Tau cohort, final model, no equality constraints on covariates | 15.301 | 11 | 0.169 | 0.061 | 0.899 |
| **Figure 3** | Add-Tau cohort, final model, equality constraints on covariates | 12.281 | 15 | 0.658 | 0.56 | 1.00 |
| **Figure 3** | ADNI cohort, final model, no equality constraints on covariates | 11.297 | 6 | 0.080 | 0.056 | 0.86 |
| **Figure 3** | ADNI cohort, final model, equality constraints on covariates | 17.172 | 14 | 0.247 | 0.066 | 0.92 |

**Table S3. Demographics Add-Tau cohort.** Regional WMH volume was not available for 7 participants. Abbreviations: ASL = arterial spin labeling; BP = blood pressure; EC = entorhinal cortex; HT = hypertension; IQR = interquartile range; MCI = mild cognitive impairment; MMSE = Mini-Mental State Examination; SD = standard deviation; WMH = white matter hyperintensity.

^a^Aβ- HT- significantly different from Aβ+ HT-

^b^Aβ- HT+ significantly different from Aβ+ HT+.

|  | **Aβ**- **HT**- | **Aβ+ HT**- | **Aβ**-­­­­­­ **HT+** | **Aβ+ HT+** |
| --- | --- | --- | --- | --- |
| **N** | 72 | 14 | 30 | 22 |
| **Age at Tau PET visit, years mean (SD) [range]** | 67.5 (8.3) [51-90] | 70.1 (7.4) [58-83] | 74.7 (7.5) [57-95] | 75.1 (8.5) [58-90] |
| **Female sex, N (%)** | 37 (51.1) | 5 (35.7) | 6 (20.0) | 4 (18.2) |
| **APOE-ε4 carriers, N (%)** | 12 (16.7) | 9 (64.3)^a^ | 3 (10.0) | 5 (22.7) |
| **Education, years mean (SD)** | 15.5 (2.7) | 16.4 (2.5) | 17.0 (2.7) | 15.5 (3.7) |
| **MCI, N (%)** | 10 (13.9) | 4 (28.6) | 12 (40.0) | 10 (45.5) |
| **MMSE, mean (SD)** | 29.4 (1.0) | 29.3 (1.1) | 29.1 (1.2) | 28.3 (1.8) |
| **Vascular Risk, mean (SD) or N (%)** |  |  |  |  |
| **Systolic BP, mmHg** | 126.7 (12.6) | 128.4 (12.2) | 135.4 (14.3) | 141.5 (12.1) |
| **Diastolic BP, mmHg** | 77.0 (7.9) | 76.5 (9.0) | 79.5 (8.0) | 80.1 (8.3) |
| **BMI** | 24.5 (3.6) | 25.2 (5.5) | 26.7 (3.4) | 25.8 (3.8) |
| **Diabetes** | 2 (2.3) | 1 (7.1) | 2 (6.7) | 1 (4.5) |
| **Current smoker** | 6 (8.3) | 3 (21.4) | 2 (6.7) | 3 (13.6) |
| **Aβ-PET and tau-PET, mean (SD)** |  |  |  |  |
| **Centiloid** | 2.8 (5.6) | 24.1 (18.7)^a^ | 0.3 (6.5) | 37.4 (26.0)^b^ |
| **EC tau-PET SUVr** | 1.01 (0.21) | 0.93 (0.10) | 1.03 (0.15) | 1.1 (0.33) |
| **WMH volume, ml, median (IQR)** |  |  |  |  |
| **Occipital** | 1.3 (0.7-2.0) | 1.2 (0.7-2.8) | 2.3 (1.7-3.4) | 2.9 (1.3-3.7) |
| **Parietal** | 0.6 (0.3-2.0) | 1.0 (0.4-2.1) | 2.4 (0.8-5.1) | 2.4 (0.8-6.2) |
| **Deep Frontal** | 0 (0-0.1) | 0.1 (0-0.2) | 0.1 (0-0.4) | 0.2 (0-0.5) |
| **N (%) with ASL MRI** | 38 (52.7) | 11 (78.6) | 18 (60.0) | 11 (50.0) |

**Table S4. Demographics ADNI cohort.** Abbreviations: BP = blood pressure; EC = entorhinal cortex; HT = hypertension; IQR = interquartile range; MCI = mild cognitive impairment; MMSE = Mini-Mental State Examination; SD = standard deviation; WMH = white matter hyperintensity.

^a^Aβ- ­­­­­­HT- significantly different from Aβ+ HT-­­­­­­.

^b^Aβ- ­­­­­­HT+ significantly different from Aβ+ HT+.

|  | **Aβ**-­­­­­­ **HT**-­­­­­­ | **Aβ+ HT**- ­­­­­­ | **Aβ**- ­­­­­­**HT+** | **Aβ+ HT+** |
| --- | --- | --- | --- | --- |
| **N** | 126 | 129 | 127 | 105 |
| **Age at Tau PET visit, years mean (SD) [range]** | 70.7 (7.5) [51 −­­­­­­ 90] | 73.5 (7.3) [57 −­­­­­­ 92]^a^ | 73.5 (8.1) [53 −­­­­ 92] | 75.3 (7.3) [58 −­­­­­­ 93] |
| **Female sex, N (%)** | 94 (58.0) | 66 (51.1) | 55 (43.3) | 60 (57.1)^b^ |
| **APOE-ε4 carriers, N (%)** | 31 (19.1) | 67 (51.9)^a^ | 27 (21.3) | 54 (51.4)^b^ |
| **Education, years mean (SD)** | 16.7 (2.3) | 16.8 (2.3) | 16.3 (2.8) | 16.3 (2.4) |
| **MCI, N (%)** | 47 (29.0) | 55 (42.6)^a^ | 46 (36.2) | 44 (41.9) |
| **MMSE, mean (SD)** | 28.9 (1.3) | 28.3 (2.0)^a^ | 28.6 (1.6) | 28.5 (1.8) |
| **Vascular Risk, mean (SD)** |  |  |  |  |
| **Systolic BP, mmHg** | 128.7 (14.0) | 128.9 (16.8) | 137.3 (15.7) | 139.3 (19.5) |
| **Diastolic BP, mmHg** | 73.9 (8.5) | 72.7 (10.3) | 75.0 (8.6) | 75.5 (9.5) |
| **BMI** | 27.2 (5.3) | 26.7 (6.6) | 28.6 (4.4) | 28.8 (6.1) |
| **Aβ-PET and tau-PET, mean (SD)** |  |  |  |  |
| **Centiloid** | -1.6 (8.5) | 58.4 (36.3)^a^ | -2.0 (8.8) | 59.7 (44.6)^b^ |
| **EC tau-PET SUVr** | 1.18 (0.22) | 1.50 (0.46)^a^ | 1.2 (0.34) | 1.41 (0.42) |
| **WMH volume, ml, median (IQR)** |  |  |  |  |
| **Occipital** | 0.2 (0.1-0.7) | 0.5 (0.1-1.4)^a^ | 0.4 (0.1-1.1) | 0.7 (0.2-1.3)^b^ |
| **Parietal** | 0.8 (0.3-1.9) | 1.3 (0.4-4.3)^a^ | 1.2 (0.4-3.8) | 1.8 (0.7-5.3)^b^ |
| **Deep Frontal** | 0.0 (0-0.2) | 0.1 (0-0.3)^a^ | 0.1 (0-0.4) | 0.1 (0-0.4) |
| **N (%) with ASL MRI** | 29 (23.0) | 18 (14.0) | 29 (22.8) | 13 (12.4) |

**Table S5. Demographics Add-Tau ASL subsample.** Regional WMH volume was not available for 5 participants (Aβ- HT-: n=1; Aβ+ HT-: n=1; Aβ- HT+: n=3). Abbreviations: ASL = arterial spin labeling; BP = blood pressure; EC = entorhinal cortex; HT = hypertension; IQR = interquartile range; MCI = mild cognitive impairment; MMSE = Mini-Mental State Examination; SD = standard deviation; WMH = white matter hyperintensity.

^a^Aβ- HT- significantly different from Aβ+ HT-.

^b^Aβ- HT+ significantly different from Aβ+ HT+.

|  | **Aβ**- **HT**- | **Aβ+ HT**- | **Aβ**- **HT+** | **Aβ+ HT+** |
| --- | --- | --- | --- | --- |
| **N** | 38 | 11 | 18 | 11 |
| **Age at Tau PET visit, years mean (SD) [range]** | 69.9 (8.5) [51-90] | 71.0 (8.1) [58-83] | 76.4 (8.2) [57-95] | 75.8 (9.1) [65-90] |
| **Female sex, N (%)** | 20 (52.6) | 5 (45.5) | 3 (16.7) | 1 (9.1) |
| **APOE-ε4 carriers, N (%)** | 7 (18.4) | 6 (54.5)^a^ | 1 (5.6) | 1 (9.1) |
| **Education, years mean (SD)** | 15.3 (2.7) | 15.8 (2.4) | 17.8 (2.3) | 15.7 (3.5) |
| **MCI, N (%)** | 6 (15.7) | 4 (36.4) | 7 (38.7) | 4 (36.4) |
| **MMSE, mean (SD)** | 29.2 (1.2) | 29.5 (0.7) | 29.4 (1.1) | 28.9 (0.8) |
| **Vascular Risk, mean (SD) or N (%)** |  |  |  |  |
| **Systolic BP, mmHg** | 127.4 (15.1) | 128.3 (13.6) | 137.2 (13.8) | 142.4 (13.7) |
| **Diastolic BP, mmHg** | 76.9 (9.0) | 75.0 (9.4) | 79.3 (8.0) | 79.7 (9.1) |
| **BMI** | 24.3 (3.0) | 24.9 (6.1) | 26.6 (3.7) | 26.3 (3.1) |
| **Diabetes** | 1 (2.6) | 1 (9.1) | 1 (5.6) | 0 (0.0) |
| **Current smoker** | 3 (7.9) | 3 (27.3) | 2 (11.1) | 1 (9.1) |
| **Aβ-PET and tau-PET, mean (SD)** |  |  |  |  |
| **Centiloid** | 3.1 (5.8) | 25.9 (20.9)^a^ | -0.2 (6.6) | 28.8 (15.8)^b^ |
| **EC tau-PET SUVr** | 0.99 (0.26) | 0.93 (0.10) | 1.05 (0.13) | 0.95 (0.12)^b^ |
| **WMH volume, ml, median (IQR)** |  |  |  |  |
| **Occipital** | 1.5 (0.8-2.0) | 1.3 (0.7-3.3) | 2.7 (1.9-3.4) | 1.8 (1.3-3.4) |
| **Parietal** | 0.6 (0.2-2.0) | 1.2 (0.6-2.8) | 1.6 (0.7-6.0) | 2.5 (1.3-5.2) |
| **Deep Frontal** | 0 (0-0.1) | 0.1 (0-0.4) | 0.1 (0-0.4) | 0.2 (0-0.8) |

**Table S6. Demographics ADNI ASL subsample.** Abbreviations: BP = blood pressure; EC = entorhinal cortex; HT = hypertension; IQR = interquartile range; MCI = mild cognitive impairment; MMSE = Mini-Mental State Examination; SD = standard deviation; WMH = white matter hyperintensity.

^a^Aβ- ­­­­­­HT−­­­­­­ significantly different from Aβ+ HT-­­­­­­.

^b^Aβ-­­­­­­ ­­­­­­HT+ significantly different from Aβ+ HT+.

|  | **Aβ**-­­­­­­ **HT**- | **Aβ+ HT**- | **Aβ**- ­­­­­­**HT+** | **Aβ+ HT+** |
| --- | --- | --- | --- | --- |
| **N** | 29 | 18 | 29 | 13 |
| **Age at Tau PET visit, years mean (SD) [range]** | 71.5 (6.2) [57 – 83] | 74 (8.5) [57 – 92] | 75.5 (8.3) [59 – 91] | 74.5 (7.6) [64 – 85] |
| **Female sex, N (%)** | 17 (58.6) | 12 (66.7) | 11 (37.9) | 7 (53.8) |
| **APOE-ε4 carriers, N (%)** | 5 (17.9) | 13 (72.2)^a^ | 8 (29.6) | 5 (41.7) |
| **Education, years mean (SD)** | 17.0 (2.0) | 16.8 (2.3) | 17 (2.9) | 16.5 (3.0) |
| **MCI, N (%)** | 7 (24.1) | 7 (38.9) | 10 (34.4) | 5 (38.5) |
| **MMSE, mean (SD)** | 29.1 (1.4) | 28 (2.3) | 28.9 (1.3) | 28.6 (1.9) |
| **Vascular Risk, mean (SD)** |  |  |  |  |
| **Systolic BP, mmHg** | 126.8 (17.4) | 135.7 (18.1) | 142.1 (19.6) | 138.7 (20.3) |
| **Diastolic BP, mmHg** | 72.9 (8.1) | 71.1 (10.1) | 75.5 (8.6) | 72.2 (9.4) |
| **BMI** | 26.4 (4.5) | 24.9 (4.5) | 28.2 (4.7) | 28.2 (6.8) |
| **Aβ-PET and tau-PET, mean (SD)** |  |  |  |  |
| **Centiloid** | 1.2 (7) | 65 (38)^a^ | 0.8 (10.1) | 69.3 (46)^b^ |
| **EC tau-PET SUVr** | 1.16 (0.12) | 1.57 (0.47)^a^ | 1.16 (0.17) | 1.44 (0.43) |
| **WMH volume, ml, median (IQR)** |  |  |  |  |
| **Occipital** | 0.3 (0.1-1.3) | 1.2 (0.4-3.0) | 1.3 (0.4-1.8) | 1.6 (0.6-3.4) |
| **Parietal** | 0.5 (0.1-3.3) | 1.2 (0.4-6.3) | 0.9 (0.2-3.1) | 1.2 (0.5-4.5) |
| **Deep Frontal** | 0.1 (0-0.2) | 0.1 (0-1.0) | 0.1 (0-1.1) | 0.1 (0-0.9) |

**Table S7. Results of the logistic regression models examining hypertension as a predictor of Aβ positivity.**

|  | **Add-Tau** | | **ADNI** | |
| --- | --- | --- | --- | --- |
|  | Estimate | *P* value | Estimate | *P* value |
| Intercept | -5.214 | 0.012 | -5.195 | <0.001 |
| Hypertension | 1.309 | 0.008 | -0.117 | 0.547 |
| Age | 0.035 | 0.219 | 0.0625 | <0.001 |
| Sex (male) | 0.870 | 0.099 | -0.219 | 0.259 |
| APOE4 | 1.975 | <0.001 | 1.621 | <0.001 |

**Table S8. Results of the linear regression models examining Aβ positivity as predictor of regional rWMH burden.**

|  | **Add-Tau** | | **ADNI** | |
| --- | --- | --- | --- | --- |
|  | **OCCIPITAL** | | | |
|  | Estimate | *P* value | Estimate | *P* value |
| Intercept | -0.018 | 0.877 | -0.245 | 0.001 |
| Aβ+ | -0.186 | 0.265 | 0.184 | 0.031 |
| Hypertension | 0.346 | 0.027 | 0.104 | 0.193 |
| Age | 0.648 | <0.001 | 0.391 | <0.001 |
| Sex (male) | 0.031 | 0.825 | 0.188 | 0.020 |
| APOE4 | -0.198 | 0.236 | 0.079 | 0.377 |
|  | **PARIETAL** | | | |
|  | Estimate | *P* value | Estimate | *P* value |
| Intercept | 0.024 | 0.842 | -0.048 | 0.524 |
| Aβ+ | 0.075 | 0.659 | 0.185 | 0.026 |
| Hypertension | 0.118 | 0.456 | 0.138 | 0.078 |
| Age | 0.680 | <0.001 | 0.465 | <0.001 |
| Sex (male) | -0.067 | 0.647 | -0.176 | 0.025 |
| APOE4 | -0.030 | 0.861 | -0.038 | 0.661 |
|  | **DEEP FRONTAL** | | | |
|  | Estimate | *P* value | Estimate | *P* value |
| Intercept | 0.103 | 0.488 | -0.049 | 0.500 |
| Aβ+ | 0.231 | 0.256 | 0.089 | 0.272 |
| Hypertension | 0.359 | 0.061 | 0.275 | <0.001 |
| Age | 0.356 | <0.001 | 0.501 | <0.001 |
| Sex (male) | -0.431 | 0.015 | -0.262 | 0.001 |
| APOE4 | -0.002 | 0.989 | 0.033 | 0.696 |

**Table S9. Testing paths in structural equation model 1 (compare Figure 1 in main manuscript) for differences between Aβ**- **and Aβ+ groups in the Add-Tau cohort.** Group differences in paths not involving rWMH burden have been tested in all models. The results did not differ in models including occipital, parietal, or deep frontal rWMH and are therefore only reported for the model involving occipital rWMH burden. The fit of the model with the constrained path was compared to the fit of a baseline model, in which all parameters are freely estimated in both groups using a likelihood ratio test. In a sensitivity analysis, the procedure was repeated after constraining the effects of covariates (except APOE4) to be the same for the Aβ- and Aβ+ groups.

|  |  | **no equality constraints on covariates** | | | **equality constraints on covariates** | | |
| --- | --- | --- | --- | --- | --- | --- | --- |
| **Model** | **Path constrained** | **Δ Chisq** | ***P* value** | **FDR *P***  **value** | **Δ Chisq** | ***P* value** | **FDR *P***  **value** |
| All (occipital) | Hypertension → EC Tau | **6.582** | **0.010** | **0.050** | **8.5574** | **0.003** | **0.030** |
| All (occipital) | Hypertension → EC Thickness | 0.097 | 0.754 | 0.861 | 0.0759 | 0.782 | 0.868 |
| All (occipital) | Hypertension → EM | **5.09** | **0.024** | 0.080 | **5.0785** | **0.024** | 0.120 |
| All (occipital) | EC Tau → EC Thickness | 0.0088 | 0.925 | 0.925 | 0.2027 | 0.652 | 0.868 |
| All (occipital) | EC Tau → EM | 0.0813 | 0.775 | 0.861 | 0.0889 | 0.765 | 0.868 |
| All (occipital) | EC Thickness → EM | 1.5723 | 0.209 | 0.484 | 2.762 | 0.096 | 0.320 |
|  |  |  |  |  |  |  |  |
| Occipital | Hypertension → rWMH | 1.3637 | 0.242 | 0.484 | 0.0173 | 0.895 | 0.895 |
| Occipital | rWMH → EC Thickness | **7.1322** | **0.007** | **0.050** | 0.6585 | 0.417 | 0.834 |
| Occipital | rWMH → EM | 0.3134 | 0.575 | 0.861 | 0.2546 | 0.613 | 0.868 |
| Occipital | rWMH ↔ EC Tau | 0.1816 | 0.67 | 0.861 | 2.0021 | 0.157 | 0.392 |
|  |  |  |  |  |  |  |  |
| Parietal | Hypertension → rWMH | 0.8985 | 0.343 | 0.488 | 0.4237 | 0.515 | 0.844 |
| Parietal | rWMH → EC Thickness | 0.816 | 0.366 | 0.488 | 0.1231 | 0.725 | 0.844 |
| Parietal | rWMH → EM | 0.0483 | 0.826 | 0.826 | 0.0383 | 0.844 | 0.844 |
| Parietal | rWMH ↔ EC Tau | 1.1682 | 0.279 | 0.488 | 0.2182 | 0.640 | 0.844 |
|  |  |  |  |  |  |  |  |
| Deep frontal | Hypertension → rWMH | 1.9079 | 0.167 | 0.334 | 1.7398 | 0.187 | 0.402 |
| Deep frontal | rWMH → EC Thickness | 0.0171 | 0.895 | 0.928 | 0.0117 | 0.913 | 0.988 |
| Deep frontal | rWMH → EM | 0.0079 | 0.928 | 0.928 | 0.0001 | 0.988 | 0.988 |
| Deep frontal | rWMH ↔ EC Tau | 2.8448 | 0.091 | 0.334 | 1.6342 | 0.201 | 0.402 |

**Table S10. Testing paths in structural equation model 1 (compare Figure 1 in main manuscript) for differences between Aβ- and Aβ+ groups in the ADNI cohort.** Group differences in paths not involving rWMH burden have been tested in all models. The results did not differ in models including occipital, parietal, or deep frontal rWMH and are therefore only reported for the model involving occipital rWMH burden. The fit of the model with the constrained path was compared to the fit of a baseline model, in which all parameters are freely estimated in both groups using a likelihood ratio test. In a sensitivity analysis, the procedure was repeated after constraining the effects of covariates (except APOE4) to be the same for the Aβ- and Aβ+ groups.

|  |  | **no equality constraints on covariates** | | | **equality constraints on covariates** | | |
| --- | --- | --- | --- | --- | --- | --- | --- |
| **Model** | **Path constrained** | **Δ Chisq** | ***P***  **value** | **FDR *P***  **value** | **Δ Chisq** | ***P***  **value** | **FDR *P***  **value** |
| All (occipital) | Hypertension → EC Tau | 3.5385 | 0.059 | 0.196 | 3.1485 | 0.076 | 0.250 |
| All (occipital) | Hypertension → EC Thickness | 1.8441 | 0.174 | 0.348 | 1.7065 | 0.191 | 0.382 |
| All (occipital) | Hypertension → EM | 1.0839 | 0.297 | 0.424 | 0.7441 | 0.388 | 0.548 |
| All (occipital) | EC Tau → EC Thickness | 3.815 | 0.051 | 0.196 | 2.6977 | 0.100 | 0.250 |
| All (occipital) | EC Tau → EM | **17.809** | **<0.001** | **<0.001** | **17.66** | **<0.001** | **<0.001** |
| All (occipital) | EC Thickness → EM | 0.1590 | 0.69 | 0.757 | 0.4312 | 0.511 | 0.567 |
|  |  |  |  |  |  |  |  |
| Occipital | Hypertension → rWMH | 0.09574 | 0.757 | 0.757 | 0.10192 | 0.749 | 0.749 |
| Occipital | rWMH → EC Thickness | 0.32772 | 0.567 | 0.708 | 0.5971 | 0.439 | 0.548 |
| Occipital | rWMH → EM | 2.3467 | 0.125 | **0.312** | **4.9649** | **0.0258** | 0.129 |
| Occipital | rWMH ↔ EC Tau | 1.1618 | 0.281 | 0.424 | 1.2438 | 0.264 | 0.440 |
|  |  |  |  |  |  |  |  |
| Parietal | Hypertension → rWMH | 0.0002 | 0.987 | 0.987 | 0.0834 | 0.772 | 0.772 |
| Parietal | rWMH → EC Thickness | 0.6542 | 0.418 | 0.557 | 1.3054 | 0.253 | 0.506 |
| Parietal | rWMH → EM | **4.8305** | **0.027** | **0.108** | **10.675** | **0.001** | **0.004** |
| Parietal | rWMH ↔ EC Tau | 0.7199 | 0.396 | 0.557 | 0.62884 | 0.427 | 0.569 |
|  |  |  |  |  |  |  |  |
| Deep frontal | Hypertension → rWMH | 0.63716 | 0.424 | 0.854 | 0.09658 | 0.756 | 0.758 |
| Deep frontal | rWMH → EC Thickness | 0.01143 | 0.914 | 0.914 | 0.29993 | 0.583 | 0.758 |
| Deep frontal | rWMH → EM | 0.1175 | 0.731 | 0.914 | 0.09434 | 0.758 | 0.758 |
| Deep frontal | rWMH ↔ EC Tau | 0.6286 | 0.427 | 0.854 | 0.60704 | 0.435 | 0.758 |

**Table S11. Testing paths in structural equation model 1 for differences between Aβ**- **and Aβ+ groups in the ADNI cohort excluding individuals with FLAIR image acquired on Philips Medical Systems MRI scanner (n = 70).** Group differences in paths not involving rWMH burden have been tested in all models. The results did not differ in models including occipital, parietal, or deep frontal rWMH and are therefore only reported for the model involving occipital rWMH burden. The fit of the model with the constrained path was compared to the fit of a baseline model, in which all parameters are freely estimated in both groups using a likelihood ratio test. In a sensitivity analysis, the procedure was repeated after constraining the effects of covariates (except APOE4) to be the same for the Aβ- and Aβ+ groups.

|  |  | **no equality constraints on covariates** | | **equality constraints on covariates** | |
| --- | --- | --- | --- | --- | --- |
| **Model** | **Path constrained** | **Δ Chisq** | ***P* value** | **Δ Chisq** | ***P* value** |
| All (occipital) | Hypertension → EC Tau | 3.1094 | 0.077 | 2.6519 | 0.103 |
| All (occipital) | Hypertension → EC Thickness | 1.366 | 0.242 | 1.2885 | 0.256 |
| All (occipital) | Hypertension → EM | 1.3867 | 0.239 | 0.9506 | 0.329 |
| All (occipital) | EC Tau → EC Thickness | 3.1156 | 0.077 | 2.2663 | 0.132 |
| All (occipital) | EC Tau → EM | **25.769** | **<0.001** | **18.518** | **<0.001** |
| All (occipital) | EC Thickness → EM | 0.06279 | 0.802 | 0.5331 | 0.465 |
|  |  |  |  |  |  |
| Occipital | Hypertension → rWMH | 0.14482 | 0.703 | 0.15808 | 0.690 |
| Occipital | rWMH → EC Thickness | 0.52939 | 0.466 | 1.0621 | 0.302 |
| Occipital | rWMH → EM | 2.5295 | 0.111 | **5.2109** | **0.022** |
| Occipital | rWMH ↔ EC Tau | 1.183 | 0.276 | 1.3575 | 0.244 |
|  |  |  |  |  |  |
| Parietal | Hypertension → rWMH | 0.52546 | 0.468 | 0.8739 | 0.3499 |
| Parietal | rWMH → EC Thickness | 0.24487 | 0.620 | 0.6990 | 0.4031 |
| Parietal | rWMH → EM | **3.9675** | **0.046** | **8.4238** | **0.0037** |
| Parietal | rWMH ↔ EC Tau | 0.58207 | 0.445 | 0.5517 | 0.4576 |
|  |  |  |  |  |  |
| Deep frontal | Hypertension → rWMH | 1 | 0.408 | 0.6389 | 0.424 |
| Deep frontal | rWMH → EC Thickness | 0.00810 | 0.928 | 0.3040 | 0.581 |
| Deep frontal | rWMH → EM | 0.042526 | 0.836 | 0.5600 | 0.454 |
| Deep frontal | rWMH ↔ EC Tau | 0.14086 | 0.707 | 0.1553 | 0.693 |

**Table S12. Testing paths in structural equation model 3 (compare Figure 3 in main manuscript) for differences between Aβ**- **and Aβ+ groups in the Add-Tau cohort.** The fit of the model with the constrained path was compared to the fit of a baseline model, in which all parameters are freely estimated in both groups using a likelihood ratio test. In a sensitivity analysis, the procedure was repeated after constraining the effects of covariates (except APOE4) to be the same for the Aβ- and Aβ+ groups.

|  |  | **no equality constraints on covariates** | | | **equality constraints on covariates** | | | |
| --- | --- | --- | --- | --- | --- | --- | --- | --- |
| **Model** | **Path constrained** | **Δ Chisq** | ***P* value** | **FDR *P***  **value** | | **Δ Chisq** | ***P* value** | **FDR *P***  **value** |
| MTL | Hypertension → EC Tau | 0.0660 | 0.797 | 0.987 | | 0.0850 | 0.770 | 0.989 |
| MTL | Hypertension → MTL CBF | 0.0002 | 0.987 | 0.987 | | 0.0509 | 0.821 | 0.989 |
| MTL | MTL CBF → EC Tau | 0.1305 | 0.717 | 0.987 | | 0.0001 | 0.989 | 0.989 |

**Table S13. Testing paths in structural equation model 3 (compare Figure 3 in main manuscript) for differences between Aβ**- **and Aβ+ groups in the ADNI cohort.** The fit of the model with the constrained path was compared to the fit of a baseline model, in which all parameters are freely estimated in both groups using a likelihood ratio test. In a sensitivity analysis, the procedure was repeated after constraining the effects of covariates (except APOE4) to be the same for the Aβ- and Aβ+ groups.

|  |  | **no equality constraints on covariates** | | | **equality constraints on covariates** | | |
| --- | --- | --- | --- | --- | --- | --- | --- |
| **Model** | **Path constrained** | **Δ Chisq** | ***P* value** | **FDR *P***  **value** | **Δ Chisq** | ***P* value** | **FDR *P***  **value** |
| MTL | Hypertension → EC Tau | 0.34983 | 0.5542 | 0.554 | 0.54969 | 0.4584 | 0.458 |
| MTL | Hypertension → MTL CBF | 0.84147 | 0.359 | 0.538 | 0.71346 | 0.3983 | 0.458 |
| MTL | MTL CBF → EC Tau | **7.6384** | **0.0057** | **0.017** | **7.0557** | **0.0079** | **0.023** |

**Table S14. Summary of findings.** The table uses color coding to represent the consistency of results across the two cohorts: red indicates inconsistent results, blue indicates partially consistent results, and green indicates consistent results.

| **Predictor** | **Dependent Variable** | **Add-Tau** | **ADNI** |
| --- | --- | --- | --- |
| **Hypertension** | Amyloid | Positive association | No association |
|  | EC tau | - Positive association in Aβ+  - Tendency in Aβ- | - Negative Association in Aβ+  - BUT: no sig. difference between Aβ groups |
|  | EC thickness | Positive Association | No Association |
|  | Memory | No direct association | No direct association |
|  | WMH | - No association with parietal WMH  - Positive association with occipital WMH | - No association with occipital WMH  - Positive association with parietal WMH |
|  |  | Association with deep frontal WMH | Association with deep frontal WMH |
|  | CBF | - No association with occipital, parietal, and MTL CBF  - Negative association with frontal CBF | - No association with occipital, parietal, frontal, and MTL CBF  - Negative association between SBP and MTL CBF |
|  |  | Observed negative association in all ROIs | Observed negative association in all ROIs |
| **Amyloid** | WMH | No association with occipital and parietal WMH | Positive Association with occipital and parietal WMH (full sample, not accounting for CBF) |
|  |  | No association with frontal WMH | No association with frontal WMH |
|  | CBF | No association with occipital CBF | Negative association with occipital CBF |
|  |  | No association with parietal and frontal CBF | No association with parietal and frontal CBF |
| **Regional WMH** | EC tau | No association | No association |
|  | EC thickness | - Negative association with occipital WMH in Aβ+  - No association with parietal and deep frontal WMH | - Negative association with occipital, parietal, and deep frontal WMH |
|  | Memory | No direct association | - Negative direct association deep frontal WMH  - Negative direct association in Aβ+ with parietal WMH |
| **Regional CBF** | WMH | Negative association with occipital WMH | No association with occipital WMH |
|  |  | Negative association with parietal WMH | Negative association with parietal WMH |
|  |  | No association with deep frontal WMH | No association with deep frontal WMH |
|  | EC tau | Negative association with MTL CBF (driven by Aβ+) | Negative association with MTL CBF in Aβ+ |

**Figure S1. Inclusion/exclusion pathway and sample sizes for the analyses per cohort.**





**Figure S2. Normalizing WMH volume by regional white matter (WM) volume and total intracranial volume (ICV) results in highly similar rWMH burden. A-C** show the correlations between regional WM volumes and the total ICV. **D-F** illustrate the correlations between the log-transformed regional WMH volumes normalized by regional WM volume and the log-transformed regional WMH volumes normalized by ICV.


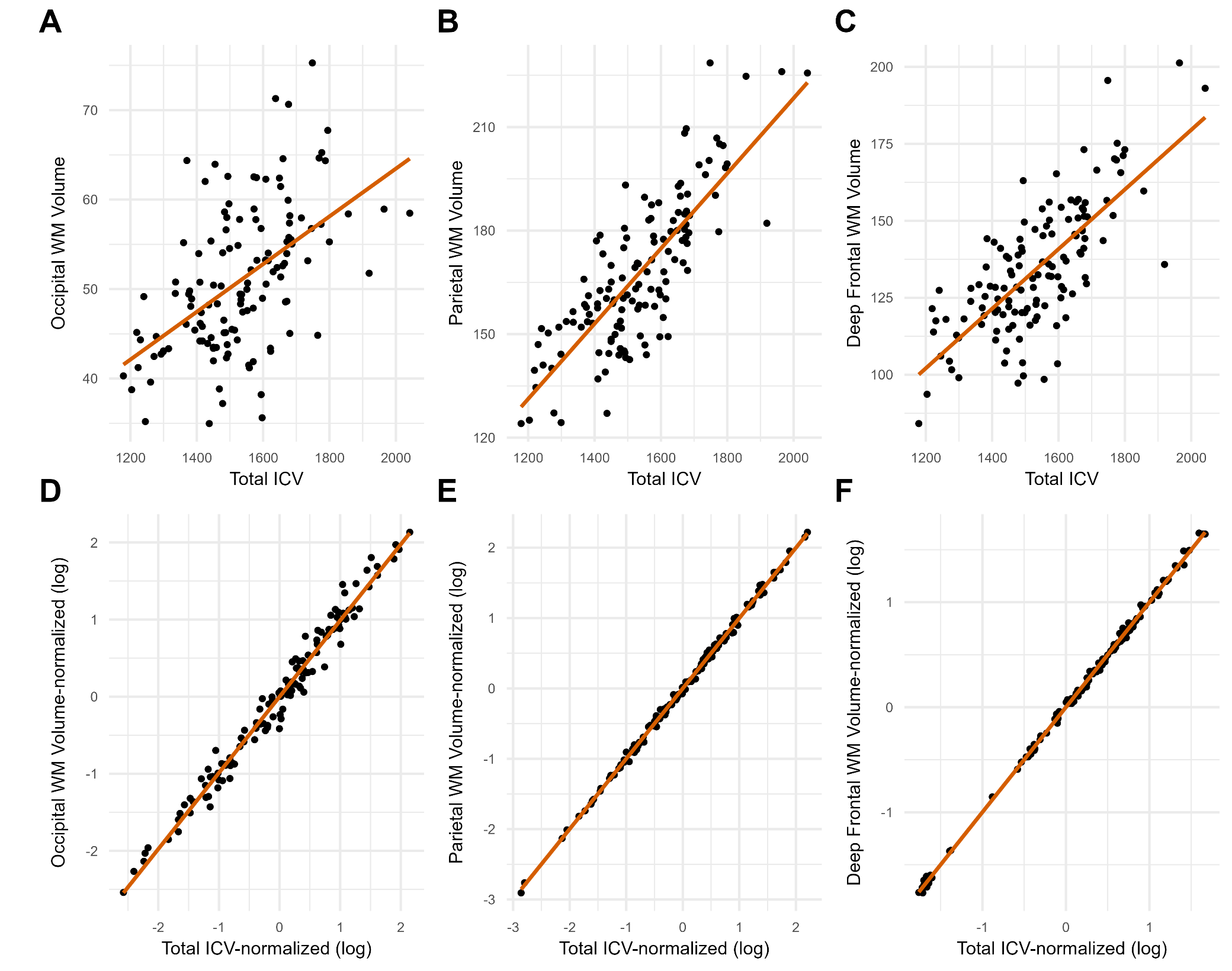


**Figure S3.** **Associations among hypertension, amyloid burden, regional CBF, and regional WMHs when CBF in the precentral gyrus was used as the CBF reference region.** The values in the boxes indicate path coefficients with a 95% confidence interval in brackets and a significance level of **P* < 0.05 and ***P* < 0.01.


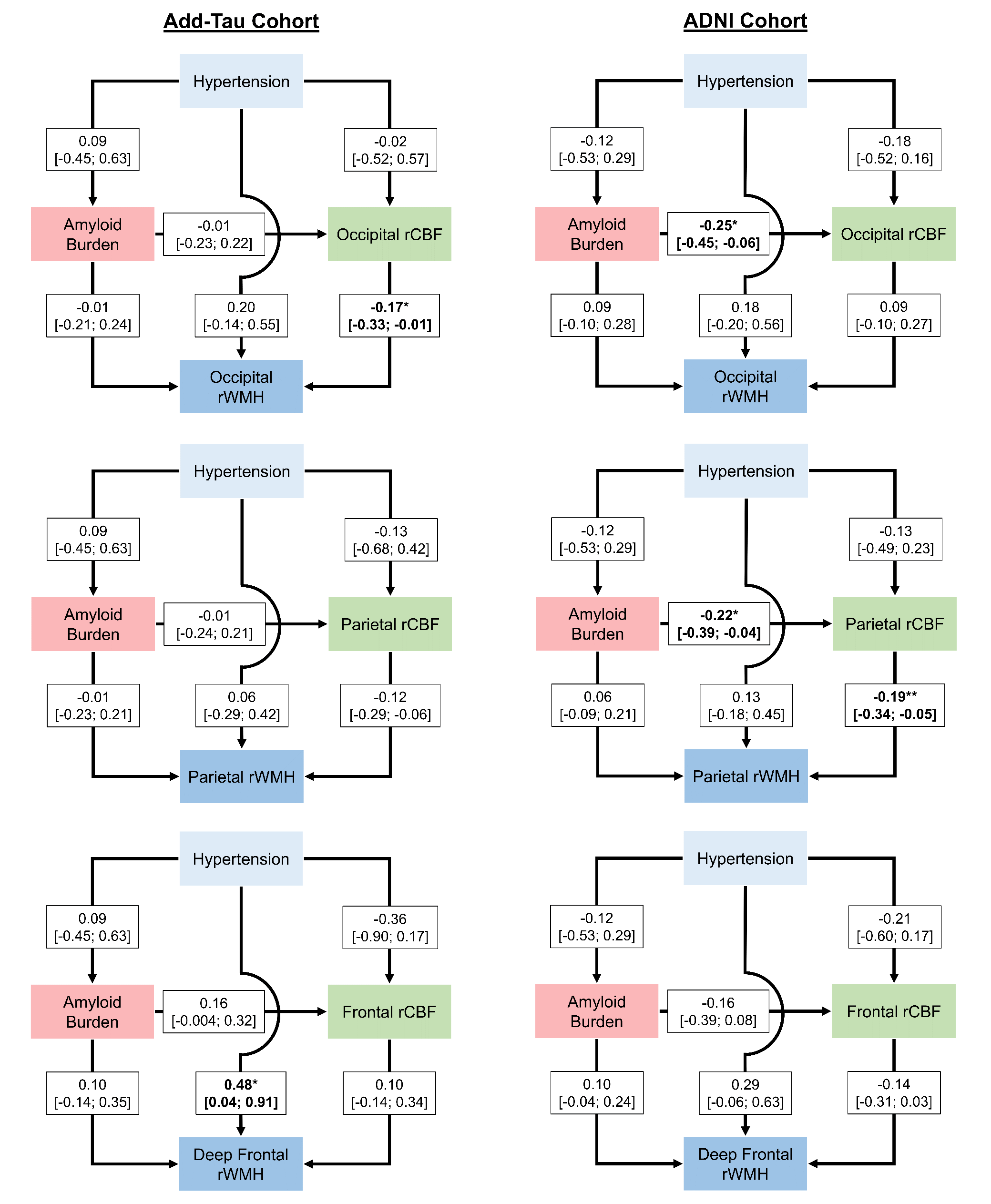


**Figure S4. Associations among hypertension, MTL CBF, and EC tau when CBF in the precentral gyrus was used as the CBF reference region.** The values in the boxes indicate path coefficients with a 95% confidence interval in brackets and a significance level of **P* < 0.05.


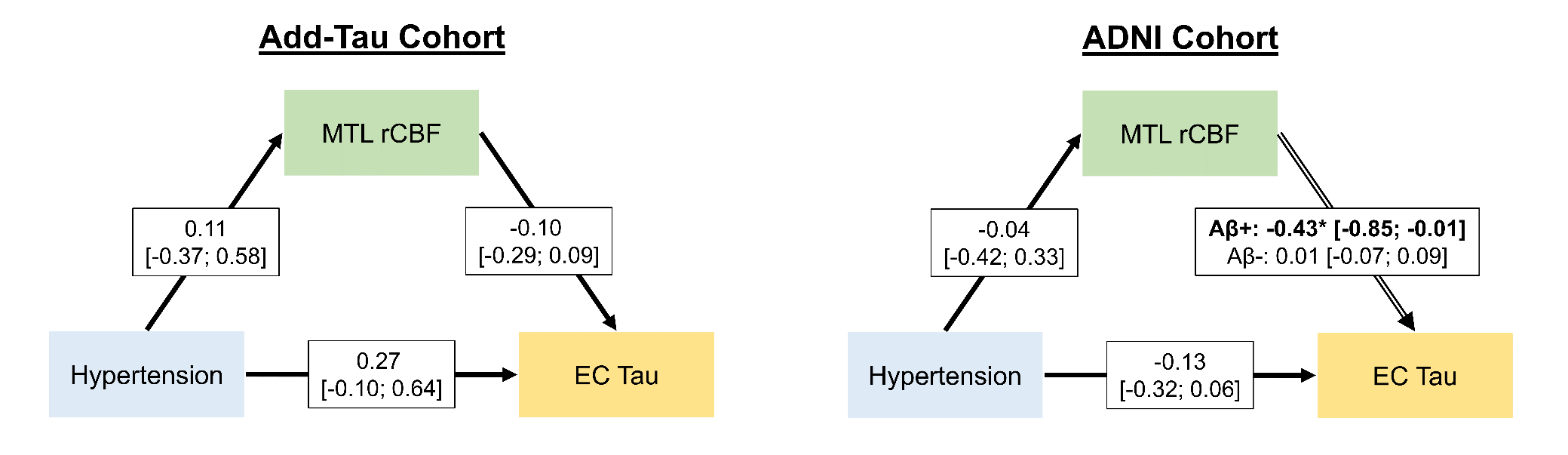


**Figure S5. Results of the final structural equation model for parietal rWMH burden.** The left-right-headed arrow indicates the residual covariance between occipital rWMH burden and EC tau. The values in the boxes indicate path coefficients with a 95% confidence interval in brackets and a significance level of **P* < 0.05, ***P* < 0.01 and ****P* < 0.001. Path coefficients were estimated separately for the low Aβ (Aβ-­­­­­­) and high Aβ (Aβ+) group for paths, which showed significant model fit decreases when constrained to be equal.


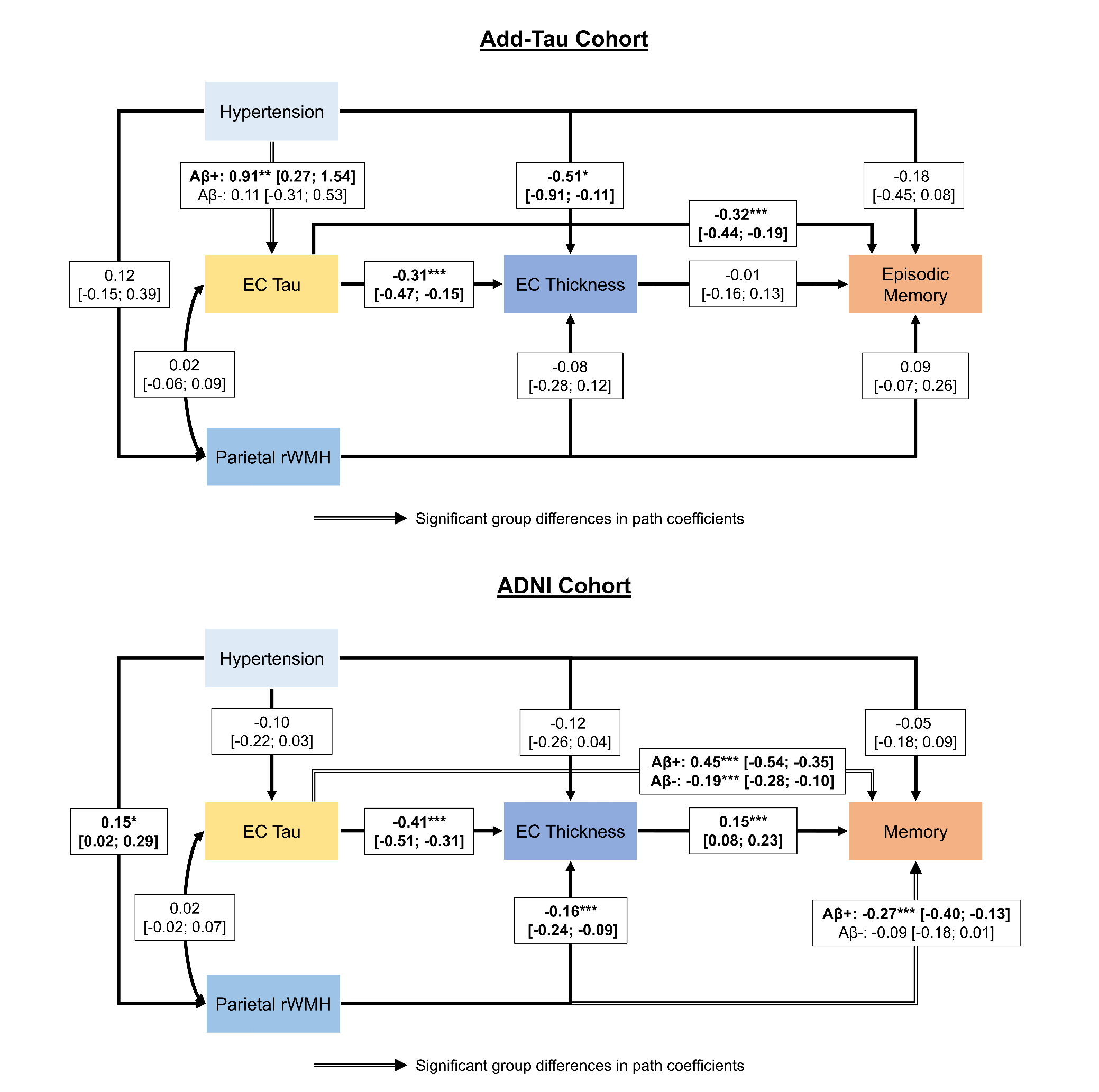


**Figure S6. Results of the final structural equation model for deep frontal rWMH burden.** The left-right-headed arrow indicates the residual covariance between occipital rWMH burden and EC tau. The values in the boxes indicate path coefficients with a 95% confidence interval in brackets and a significance level of **P* < 0.05, ***P* < 0.01 and ****P* < 0.001. Path coefficients were estimated separately for the low Aβ (Aβ-­­­­­­) and high Aβ (Aβ+) group for paths, which showed significant model fit decreases when constrained to be equal.


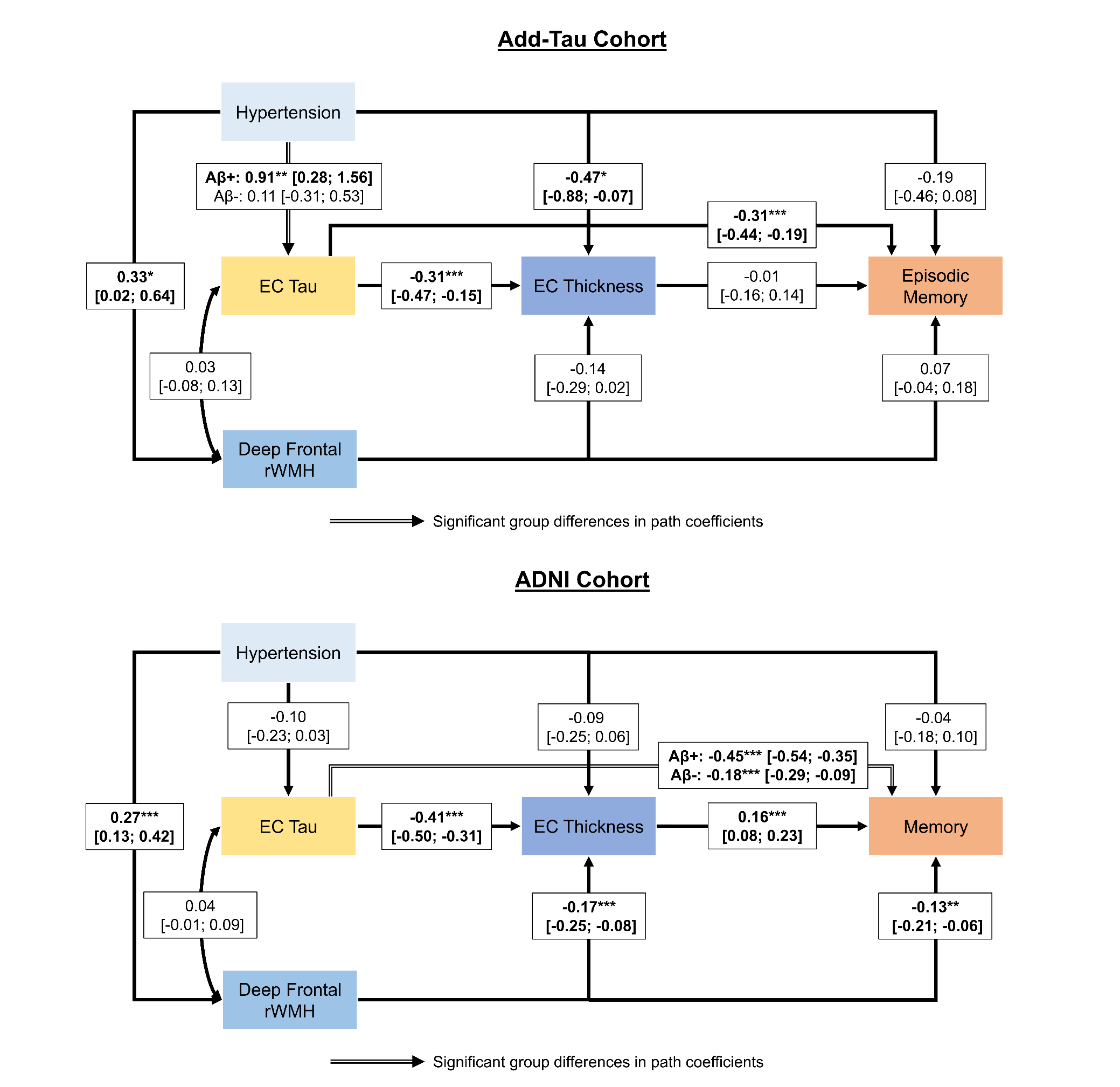


**Figure S7. Results of the final structural equation model for occipital (A), parietal (B), and (C) deep frontal rWMH burden in the ADNI cohort after excluding individuals for which the FLAIR image was acquired on Philips Medical Systems MRI scanner (n = 70).** The left-right-headed arrow indicates the residual covariance between occipital rWMH burden and EC tau. The values in the boxes indicate path coefficients with a 95% confidence interval in brackets and a significance level of **P* < 0.05, ***P* < 0.01 and ****P* < 0.001. Path coefficients were estimated separately for the low Aβ (Aβ-­­­­­­) and high Aβ (Aβ+) group for paths, which showed significant model fit decreases when constrained to be equal.


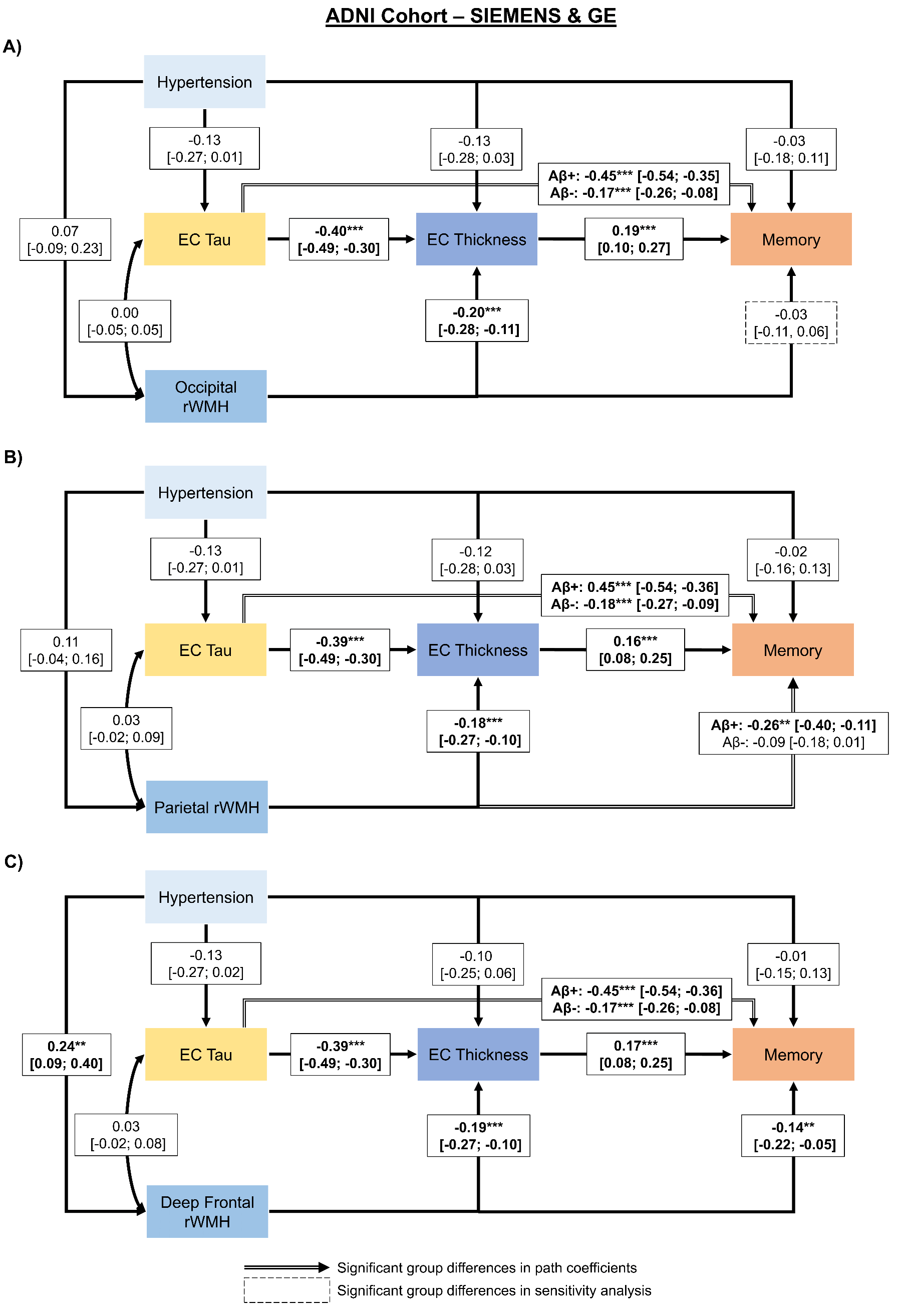


**Figure S8. Structural equation models for the Add-Tau cohort using non-PVC CBF values.** The values in the boxes indicate path coefficients with a 95% confidence interval in brackets and a significance level of **P* < 0.05, ***P* < 0.01 and ****P* < 0.001.


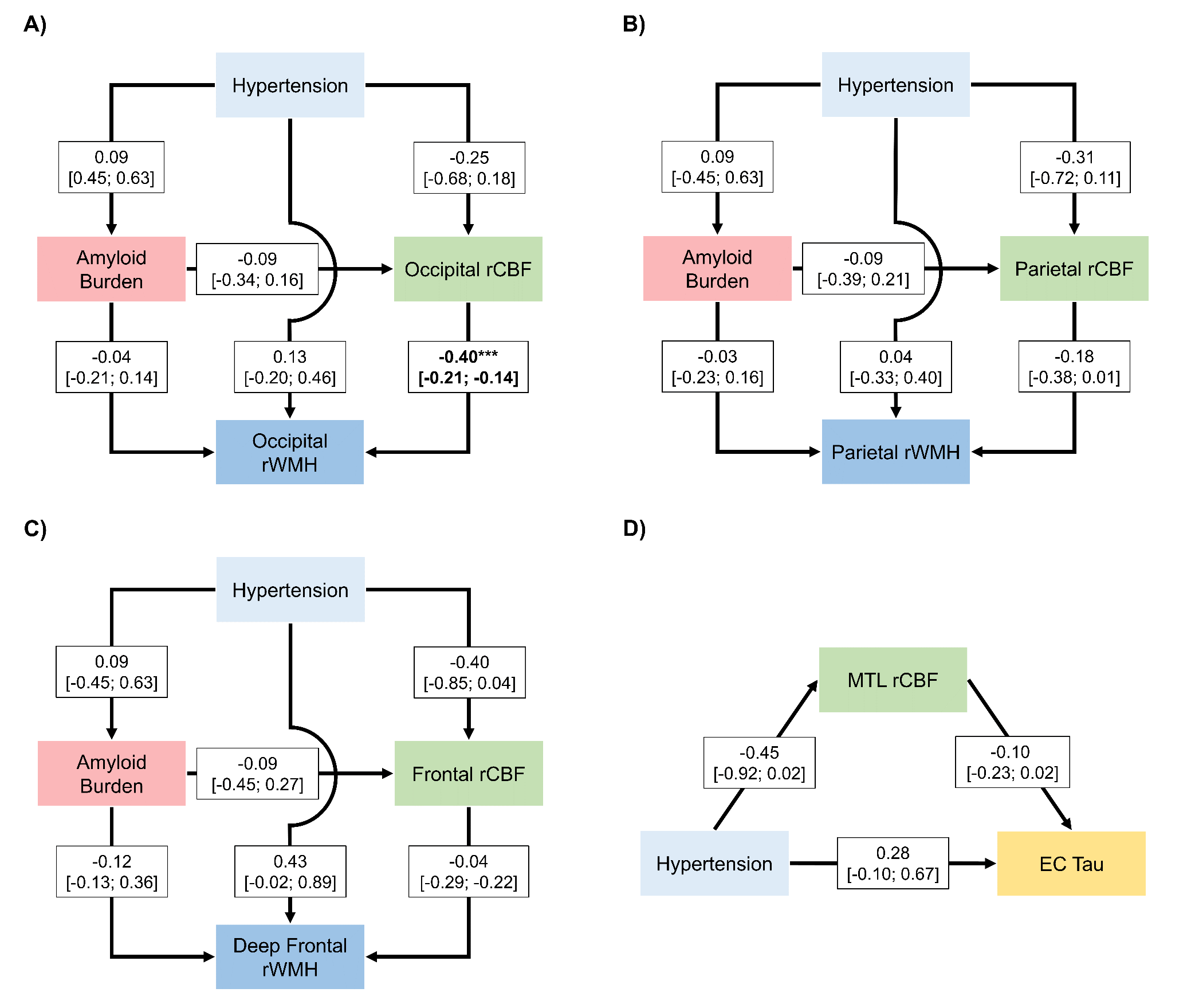


**Figure S9. Results of the structural equation models (Figure 1 in main manuscript) after replacing hypertension diagnosis with systolic blood pressure assessed at screening visits.** The left-right-headed arrow indicates the residual covariance between occipital rWMH burden and EC tau. The values in the boxes indicate path coefficients with a 95% confidence interval in brackets and a significance level of **P* < 0.05, ***P* < 0.01 and ****P* < 0.001. Path coefficients were estimated separately for the low Aβ (Aβ-­­­­­­) and high Aβ (Aβ+) group for paths, which showed significant model fit decreases when constrained to be equal.


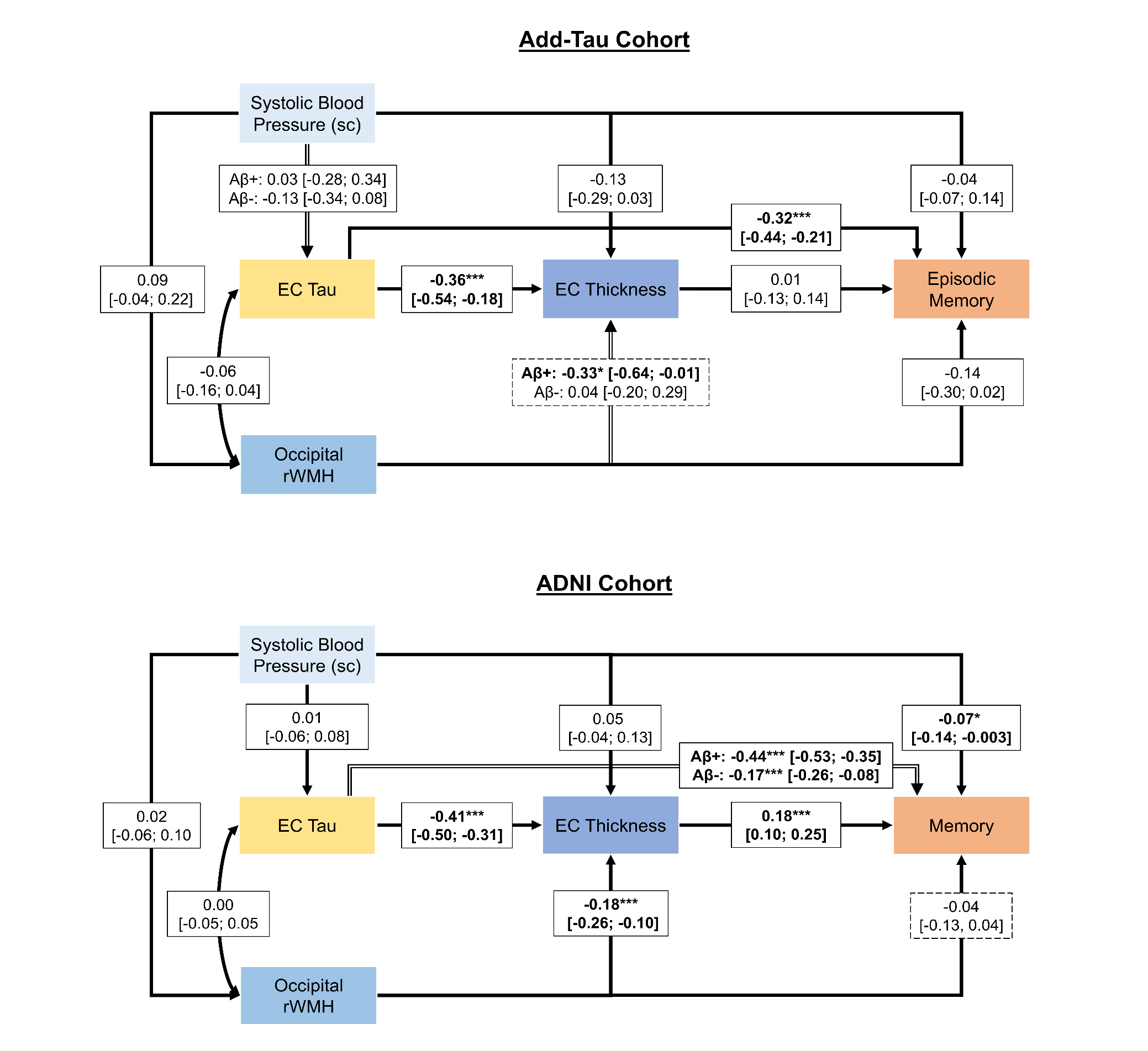


**Figure S10. Results of the structural equation models (Figure 2 in main manuscript) after replacing hypertension diagnosis with systolic blood pressure assessed at screening visits.** The values in the boxes indicate path coefficients with a 95% confidence interval in brackets and a significance level of **P* < 0.05, ***P* < 0.01 and ****P* < 0.001.


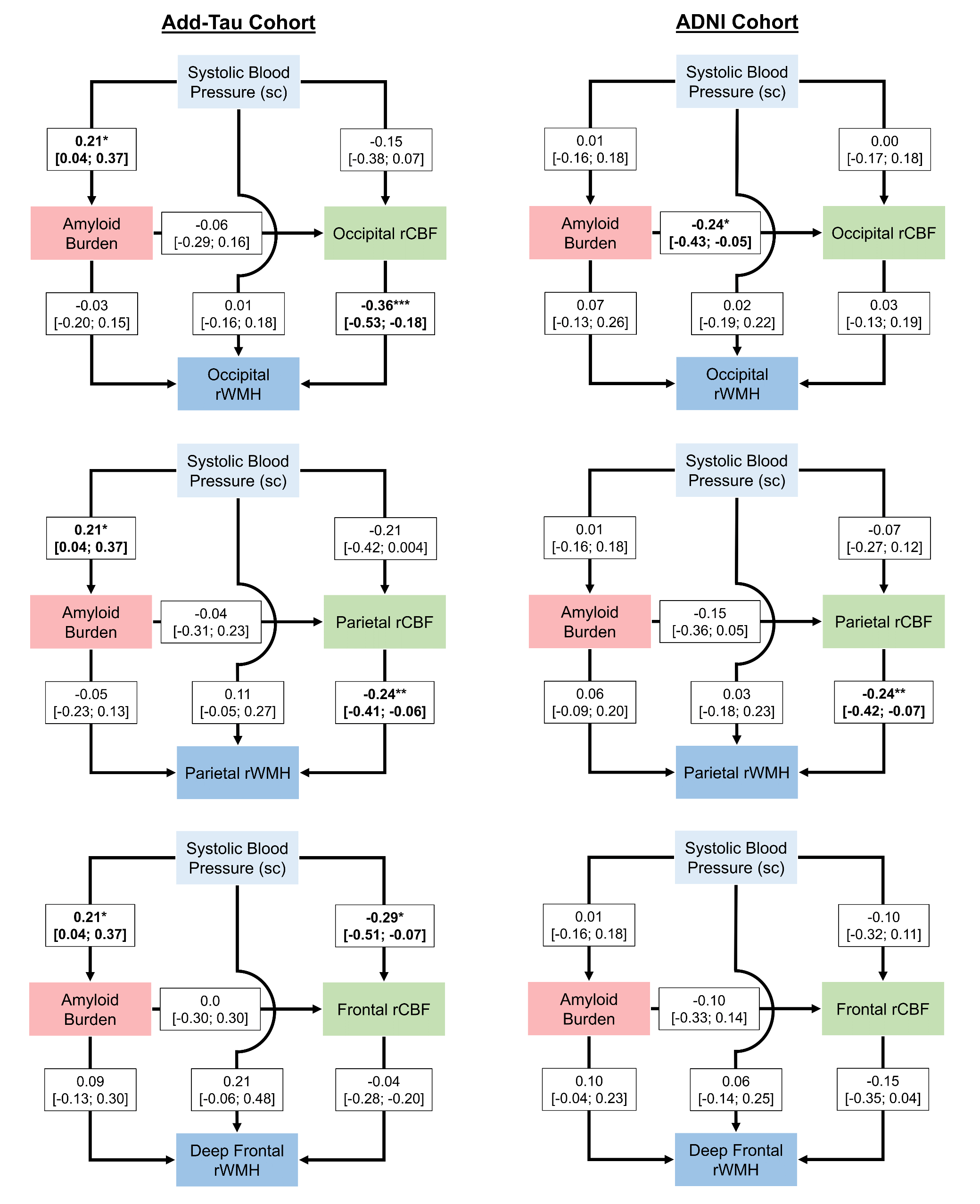


**Figure S11. Results of the moderated mediation models (Figure 3 in main manuscript) after replacing hypertension diagnosis with systolic blood pressure assessed at screening visits.** The values in the boxes indicate path coefficients with a 95% confidence interval in brackets and a significance level of **P* < 0.05, ***P* < 0.01 and ****P* < 0.001.


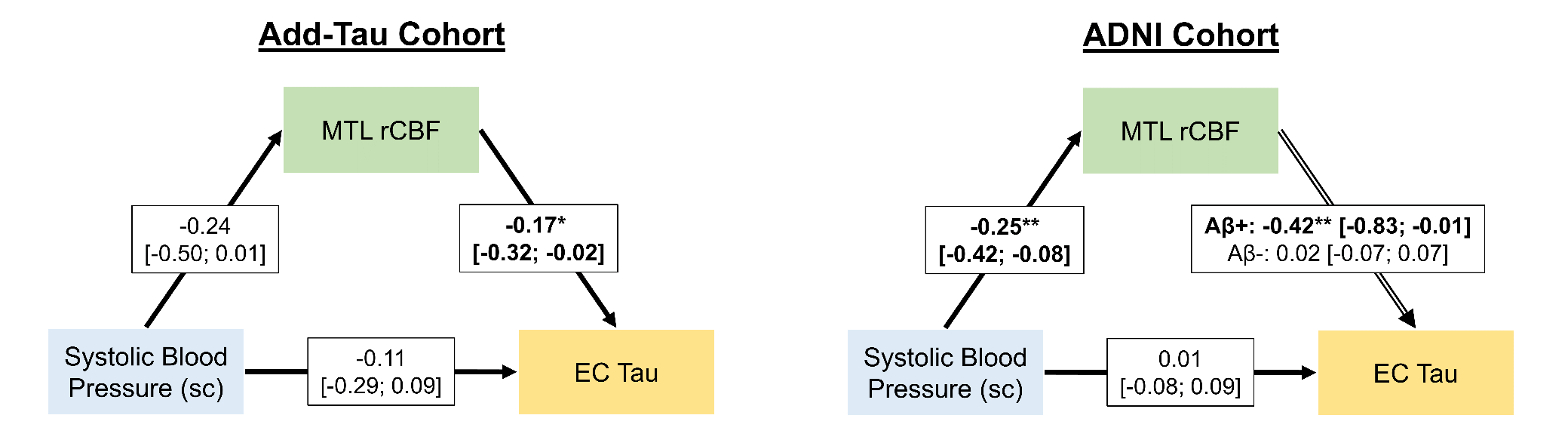


**SReferences**

1. Jenkinson M, Beckmann CF, Behrens TE, Woolrich MW, Smith SM. FSL. Neuroimage. 2012 Aug 15;62(2):782-90.

2. Greve DN, Fischl B. Accurate and robust brain image alignment using boundary-based registration. Neuroimage. 2009 Oct 15;48(1):63-72.

3. Asllani I, Borogovac A, Brown TR. Regression algorithm correcting for partial volume effects in arterial spin labeling MRI. Magnetic Resonance in Medicine. 2008;60(6):1362-71.

4. Chappell MA, Kirk TF, Craig MS, et al. BASIL: A toolbox for perfusion quantification using arterial spin labelling. Imaging Neuroscience. 2023;1:1-16.

5. Chappell MA, Groves AR, MacIntosh BJ, Donahue MJ, Jezzard P, Woolrich MW. Partial volume correction of multiple inversion time arterial spin labeling MRI data. Magnetic Resonance in Medicine. 2011;65(4):1173-83.

6. Chappell MA, McConnell FAK, Golay X, et al. Partial volume correction in arterial spin labeling perfusion MRI: A method to disentangle anatomy from physiology or an analysis step too far? Neuroimage. 2021 Sep;238:118236.

7. Alsop DC, Detre JA, Golay X, et al. Recommended implementation of arterial spin-labeled perfusion MRI for clinical applications: A consensus of the ISMRM perfusion study group and the European consortium for ASL in dementia. Magn Reson Med. 2015 Jan;73(1):102-16.

8. Klein A, Tourville J. 101 Labeled Brain Images and a Consistent Human Cortical Labeling Protocol. Frontiers in Neuroscience. 2012 2012-December-05;6.

9. Jaganmohan D, Pan S, Kesavadas C, Thomas B. A pictorial review of brain arterial spin labelling artefacts and their potential remedies in clinical studies. Neuroradiol J. 2021 Jun;34(3):154-68.

10. Schermelleh-Engel K, Moosbrugger H, Müller H. Evaluating the fit of structural equation models: Tests of significance and descriptive goodness-of-fit measures. Methods of psychological research online. 2003;8(2):23-74.

11. Shi D, Lee T, Maydeu-Olivares A. Understanding the model size effect on SEM fit indices. Educational and psychological measurement. 2019;79(2):310-34.

12. Kenny DA, McCoach DB. Effect of the Number of Variables on Measures of Fit in Structural Equation Modeling. Structural Equation Modeling: A Multidisciplinary Journal. 2003 2003/07/01;10(3):333-51.

13. Wolf EJ, Harrington KM, Clark SL, Miller MW. Sample Size Requirements for Structural Equation Models: An Evaluation of Power, Bias, and Solution Propriety. Educ Psychol Meas. 2013 Dec;76(6):913-34.
